# Non‐Precious Metal Catalysts with Gradient Oxidative Dual Sites Boost Bimolecular Activation for Catalytic Oxidation Reactions

**DOI:** 10.1002/anie.202506018

**Published:** 2025-04-21

**Authors:** Yufei Wang, Tianwei Lan, Lupeng Han, Evangelina Pensa, Yongjie Shen, Xingchi Li, Zixiang Xu, Xin Chen, Mengxue Wang, Xiaoya Xue, Yanqing Li, Ming Xie, Emiliano Cortés, Dengsong Zhang

**Affiliations:** ^1^ International Joint Laboratory of Catalytic Chemistry State Key Laboratory of Advanced Special Steel Innovation Institute of Carbon Neutrality Department of Chemistry College of Sciences Shanghai University Shanghai 200444 P.R. China; ^2^ Nanoinstitute Munich Faculty of Physics Ludwig‐Maximilians‐Universität (LMU) 80539 Munich Germany; ^3^ Institute for Chemical Reaction Design and Discovery (WPI‐ICReDD) Hokkaido University Sapporo 001–0021 Japan; ^4^ Department of Chemical Engineering University of Bath Bath BA27AY UK

**Keywords:** Ammonia oxidation, Catalytic interface, Dual sites, Imide mechanism

## Abstract

Catalytic oxidation emerges as a highly promising and cost‐effective approach for eliminating gaseous pollutants, greenhouse gases, and volatile organic compounds (VOCs) from industrial exhaust streams. However, achieving the simultaneous activation of O_2_ and substrate molecules at low temperatures using non‐precious metal catalysts remains a significant challenge. In this study, we introduce gradient oxidative Cu─O─Ti/Cu─O─Cu dual sites that enhance bimolecular activation for catalytic oxidation reactions. The catalyst, Ti‐doped CuO, is synthesized on a TiO_2_ support through the immobilization of Cu^2⁺^ on NO_3_⁻‐grafted TiO_2_, followed by thermal treatment. The resulting gradient oxidative Cu─O─Ti/Cu─O─Cu sites exhibit exceptional catalytic oxidation activity for NH_3_ and various VOCs at low temperatures, matching the performance of precious metal‐based catalysts. Notably, during NH₃ oxidation, Cu─O─Ti sites enhance the activation of both O₂ and NH₃. HNO intermediates formed on Cu─O─Ti sites react with NH intermediates on neighboring Cu─O─Cu sites—producing N₂ and H₂O via an imide mechanism—which effectively lowers the reaction barrier for catalytic NH₃ oxidation. As such, dual sites in non‐precious metal catalysts show promising results for advancing future catalytic oxidation technologies.

## Graphical abstract

Engineering highly active dual sites enhances catalytic oxidation reactions by optimizing bimolecular activation. By immobilizing Cu^2+^ on NO_3_⁻‐grafted TiO_2_ and applying thermal treatment, Ti‐doped CuO is successfully synthesized on a TiO_2_ support. This catalyst, featuring gradient oxidative Cu─O─Ti/Cu─O─Cu dual sites, shows strong potential for low‐temperature catalytic removal of NH₃ and various volatile organic compounds (VOCs) for environmental applications.

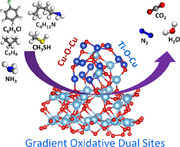

## Introduction

Catalytic oxidation stands as an exceptionally potential and cost‐effective method for the elimination of gaseous pollutants, greenhouse gases, and volatile organic compounds (VOCs) from industrial exhaust streams.^[^ ^1^, ^2^, ^3^ ^]^ The catalytic oxidation performance of catalysts depends on the adsorption and activation ability of O_2_ and substrate molecules, which also results in diverse reactive intermediates and reaction pathways.^[^ ^4^ ^]^ Generally, precious metal‐based catalysts, such as Pt,^[^ ^5^ ^]^ Pd,^[^ ^6^ ^]^ and Ag,^[^ ^7^ ^]^ exhibit high catalytic oxygen activity, attributed to their superior capacity for activating O_2_. Considering the cost of catalysts in large‐scale applications, it is imperative to develop non‐precious metal‐based catalysts as alternatives. However, it is a significant hurdle to achieve a simultaneous activation of O_2_ and substrate molecules using non‐precious metal‐based catalysts, leading to inferior catalytic activity at low temperatures.^[^ ^8^ ^]^ Therefore, the design of multifunctional active sites has been gradually developed, among which dual sites receive increasing interest due to their outperforming catalytic performance.^[^ ^9^ ^]^ Dual sites with different oxidation states of metals have shown distinct advantages in heterogeneous coupling and hydrogenation reactions. Abundant Ni^2+^─Ni^δ+^ dual sites endow the adjacent CO intermediates with distinct charge densities, hence lowering the C─C coupling reaction barrier.^[^ ^10^ ^]^ The Ni^0^─Ni^δ+^ dual sites also exhibit higher intrinsic activity than the single Ni^0^ or Ni^δ+^ sites in hydrogenation of nitriles to primary amines due to the facilitated activation of H_2_ and nitriles on Ni^0^ and Ni^δ+^, respectively.^[^ ^11^ ^]^ The diverse oxidation states of metal also have distinct roles in the activation of reactants for oxidation reactions. The metallic Cu has been demonstrated to be the most active phase in the CO oxidation reaction because of the favorable adsorption of active O species.^[^ ^12^ ^]^ Besides, the Cu^1+^ and Cu^0^ species have lower reaction activation energy than Cu^2+^ species.^[^ ^13^ ^]^ However, it is challenging to stably catalyze oxidation reactions because the low valent Cu^0^/Cu^δ+^ species in dual sites are generally not thermally stable in oxygen‐containing atmospheres. One possible alternative approach is constructing gradient oxidative dual sites via tuning the formation ability of oxygen vacancy of neighboring active sites to generate different O_2_ activation capacity.

Ammonia (NH_3_) as one of the gaseous pollutants, contributes to the formation of PM2.5, impairing the atmospheric environment and human health.^[^ ^14^ ^]^ NH_3_ selective catalytic oxidation (NH_3_‐SCO) is demonstrated to be an efficient and environmentally friendly NH_3_ abatement technology.^[^ ^15^ ^]^ The typical CuO‐based catalysts usually follow the i‐SCR mechanism, where NH_3_ is gradually dehydrogenated and oxidized to nitrogen oxide species that further react with NH_x_ species to produce N_2_.^[^ ^16^ ^]^ However, the intricate i‐SCR reaction pathway has sluggish kinetics and results in dissatisfactory low‐temperature activity, and T_90_ (the temperature at which NH_3_ conversion reaches 90%) always surpasses 240 °C. Designing gradient oxidative Cu‐based dual sites is expected to improve low‐temperature NH_3_ oxidation activity via promoting simultaneous activation of O_2_ and NH_3_. Herein, Ti dopped CuO was meticulously engineered and created on TiO_2_ support (Ti─CuO/TiO_2_), which possessed gradient oxidative Cu─O─Ti/Cu─O─Cu dual sites. This catalyst exhibits superior low‐temperature activity with a T_90_ of 210 °C and N_2_ selectivity of 90%, which strongly competes with precious metal‐based catalysts. The comprehensive in situ characterizations combined with DFT calculations demonstrate that Cu─O─Ti sites enhance the activation of O_2_ and NH_3_ and promote the formation of HNO intermediates that react with NH intermediates on adjacent Cu─O─Ti sites to generate N_2_ and H_2_O at low temperatures via an imide mechanism, which requires a lower reaction barrier for the NH₃ catalytic oxidation. This work offers an innovative approach to enhance catalytic oxidation reactions by designing highly active dual sites that optimize bimolecular activation and reaction pathways.

## Results and Discussion

**Figure 1:**
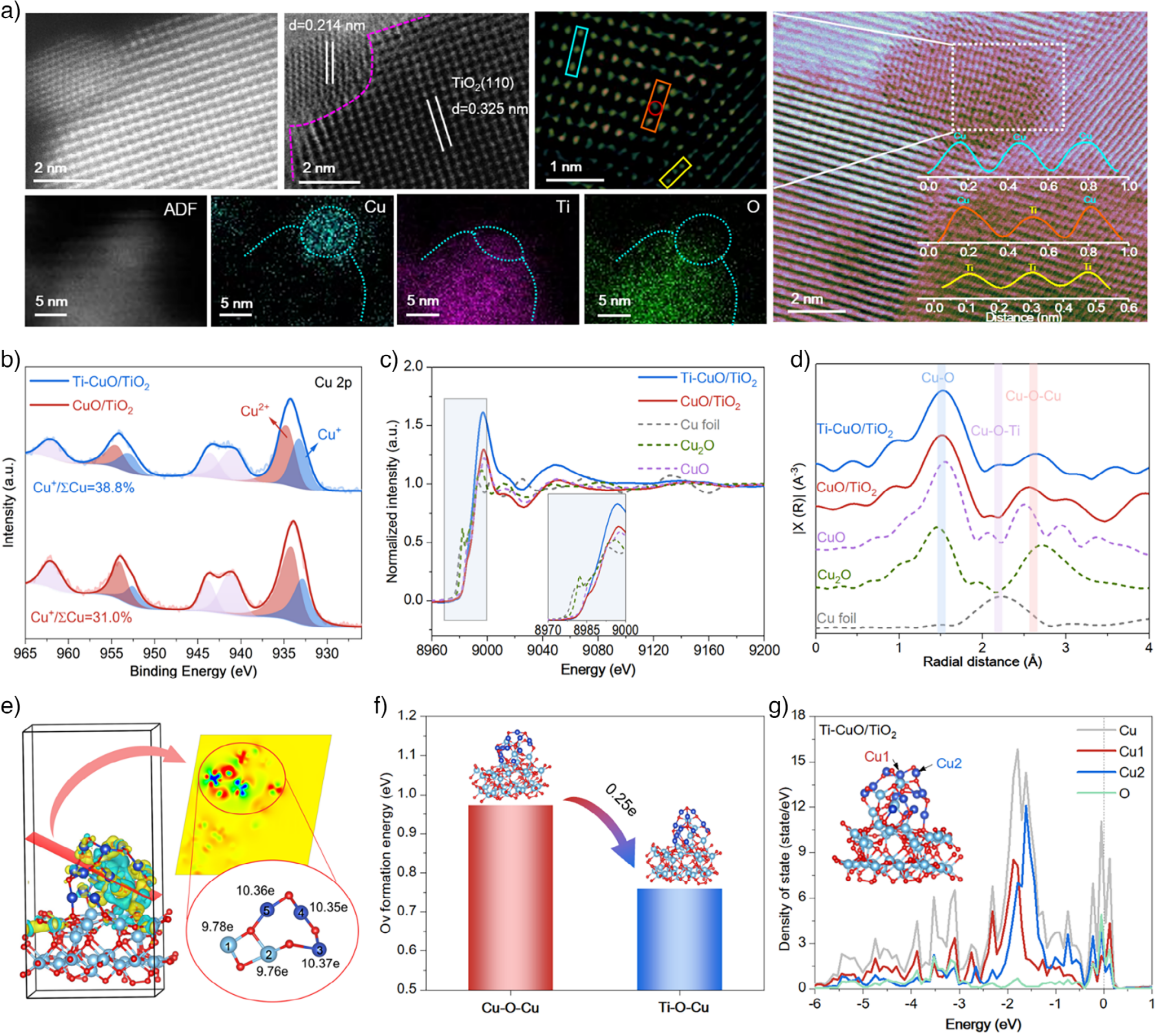
Structural characterizations. a) AC‐HAADF‐STEM pictures, inverse FFT pattern, and EDS mapping results of Ti─CuO/TiO_2_. b) Cu 2p XPS spectra of Ti─CuO/TiO_2_ and CuO/TiO_2_. c) The Cu K‐edge XANES spectra of Ti─CuO/TiO_2_ and CuO/TiO_2_ and the references (Cu foil, CuO, and Cu_2_O). d) The R‐space Cu K‐edge EXAFS spectra of Ti─CuO/TiO_2_ and CuO/TiO_2_ and the references (Cu foil, CuO, and Cu_2_O). e) Interfacial structure of 2D (right) and 3D (left) isosurfaces of local charge density difference in Ti substituted CuO (111) structure. Electron accumulation and depletion are represented by red and blue areas, respectively. f) Oxygen vacancy formation energy of Cu─O─Cu and Ti─O─Cu in Ti─CuO/TiO_2_ models. g) DOS of Cu in Cu1─O─Cu and Cu2─O─Ti of Ti─CuO/TiO_2_ models. The Fermi level (*E*_F_) is set to 0 eV. Light blue, dark blue, and red balls represented Ti, Cu, and O atoms, respectively. The CuO loading amount of Ti─CuO/TiO_2_ is 10 wt% unless otherwise specified.

The Ti─CuO/TiO_2_ catalyst with gradient oxidative Cu─O─Ti/Cu─O─Cu dual sites was synthesized through the immobilization of Cu^2+^ by NO_3_^⁻^ grafted on TiO_2_ surface followed by a thermal treatment (Figure S1). Firstly, the TiO_2_ support grafted with abundant NO_3_^⁻^ groups (TiO_2_─NA) was successfully prepared by the HNO_3_ modified sol‐gel method, evidenced by the N1s spectra of X‐ray photoelectron spectroscopy (XPS) as well as Fourier transform infrared spectroscopy (FT‐IR) spectra (Figures S2 and S3). After impregnating TiO_2_─NA with Cu^2+^ precursor followed by a high‐temperature calcination process, Ti dopped CuO was created on TiO_2_ support due to the immobilization of Cu^2+^ by NO_3_^⁻^ grafted on TiO_2_. As a comparison, CuO/TiO_2_ was also prepared using the TiO_2_ without the NO_3_^⁻^ group. From the X‐ray diffraction (XRD) patterns (Figure S4), Ti─CuO/TiO_2_ shows typical diffraction peaks of rutile TiO_2_ (JCPDS No. 21–1276) without copper‐related phases, indicating the high dispersion of supported copper species.^[^ ^17^ ^]^ As a comparison, CuO/TiO_2_ shows the characteristic diffraction peaks of CuO, implying the existence of large CuO crystals. The high‐angle annular dark‐field scanning transmission election microscope (HAADF‐STEM) images with energy‐dispersive X‐ray spectroscopy (EDS) mapping also confirm that the copper species deliver a more dispersed state on Ti─CuO/TiO_2_ than CuO/TiO_2_ (Figures S5 and S6). The copper dispersion was quantified through a dissociative N_2_O oxidation and H_2_ titration method, and the Cu dispersion (0.44) of Ti─CuO/TiO_2_ is higher than that (0.21) of CuO/TiO_2_ (Table S1). From the high‐resolution transmission electron microscope (HRTEM), CuO/TiO_2_ shows a large CuO particle with the lattice fringes of 0.235 nm, corresponding to CuO (111) (Figure S7). Interestingly, Ti─CuO/TiO_2_ shows a smaller CuO particle, with the CuO (111) lattice fringes contracting to 0.223 nm. (Figure S8). This result is likely attributed to the Ti doping into the lattice of CuO and the smaller Ti^4^⁺ ionic radius (0.67 Å) than that (0.73 Å) of Cu^2+^. Further, the aberration‐corrected HAADF‐STEM (AC‐HAADF‐STEM) was used to analyze the distribution of atoms at the interface of CuO and TiO_2_. As seen in Figure 1a, Ti─CuO/TiO_2_ also shows a decreased CuO (111) lattice fringe of 0.214 nm resulting from the contraction of the CuO lattice. The inverse fast Fourier transform (FFT) pattern shows different brightness levels for Ti and Cu atoms that exist in the bulk of CuO and TiO_2_, respectively. Interestingly, Ti and Cu appear intermingled at the interface of CuO and TiO_2_ (Figure 1a), indicating the Ti atoms have been successfully doped into the lattice of CuO. The EDS mapping illustrates that Ti is present within portions of the CuO nanoparticle (Figure 1a), also evidencing the doping of Ti at the interface between CuO and TiO_2_. The Ti doping induces the changes of electronic structure of Cu. The Cu 2p XPS spectra show a lower valence state of Cu in Ti─CuO/TiO_2_ than in CuO/TiO_2_ for the higher Cu^+^/(Cu^+^+Cu^2+^) fraction (38.8% vs 31.0%, Figure 1b and Table S2), indicating Cu in Ti─CuO/TiO_2_ presents a more electron‐rich state. The low‐valent Cu species facilitate NH_3_ chemisorption and N─H bond activation.^[^ ^18^ ^]^ Further, X‐ray absorption spectroscopy (XAS), including X‐ray absorption near‐edge structure (XANES) and extended X‐ray absorption fine structure (EXAFS), was performed to examine the electronic structure and coordination environment of Cu in Ti─CuO/TiO_2_ and CuO/TiO_2_. In the Cu K‐edge XANES spectra (Figure 1c), the absorption edge of two catalysts is similar to CuO, indicating that CuO is the main phase. It is notable that Ti─CuO/TiO_2_ shows lower energy of the white line than CuO/TiO_2_, evidencing the lower valence state of Cu over Ti─CuO/TiO_2_, in line with the XPS results. EXAFS spectra of Ti─CuO/TiO_2_ and CuO/TiO_2_ both display the peak around 1.55 Å assigned to the Cu─O (first shell) and 2.65 Å assigned to Cu─O─Cu (second shell) of CuO (Figure 1d). Differently, Ti─CuO/TiO_2_ shows the new peak around 2.25 Å, which is attributed to the Cu─O─Ti (second shell). The coordination number of Cu─O─Cu (∼1.7) in Ti─CuO/TiO_2_ is lower than that (∼3.0) in CuO/TiO_2_ (Tables S3 and S4), suggesting the higher dispersed state of Cu species on Ti─CuO/TiO_2_. Besides, the coordination number (1.0) of Cu─O─Ti is lower than that (1.7) of Cu─O─Cu in Ti─CuO/TiO_2_ (Table S3), in which the low coordinated state of Cu is more favorable for the reactant adsorption.^[^ ^18^ ^]^ DFT calculations were performed to clarify the electronic structure of Ti─CuO/TiO_2_. Based on the HRTEM results, Ti─CuO/TiO_2_ is modeled by TiO_2_ (110) supported Ti doped CuO (111) clusters, while CuO/TiO_2_ is modeled by TiO_2_ (110) supported CuO (111) clusters (Figures S9 and S10). As displayed in Figure 1e, the charge density difference analysis of Ti─CuO/TiO_2_ model indicates a significant charge redistribution at the linkage of Ti─O─Cu─O─Cu─O─Cu, where the electron density of Cu in the Cu─O─Ti segment is higher than that in the Cu─O─Cu one. This result further evidences the speculation from XPS and XANES. The bond length of Cu─O in Cu─O─Ti is also longer than that in Cu─O─Cu (Figure S11), indicating the weaker Cu─O bond strength and easier formation of oxygen vacancy.^[^ ^19^ ^]^ The DFT calculations evidence that the formation energy of oxygen vacancy in Cu─O─Ti is decreased by 0.25 compared to that in Cu─O─Cu (Figure 1f), indicating that the Cu─O─Ti structure facilitates oxygen migration ability. From the density of state ([DOS], Figure 1g), the d‐band centers (*E*_d_) energy level (−1.85 eV) of Cu in Cu─O─Ti is higher than that (−2.46 eV) in Cu─O─Cu. The upgrade of *E*_d_ energy levels of Cu in Cu─O─Ti is beneficial to enhance the adsorption and activation of reactants.^[^ ^20^ ^]^

**Figure 2:**
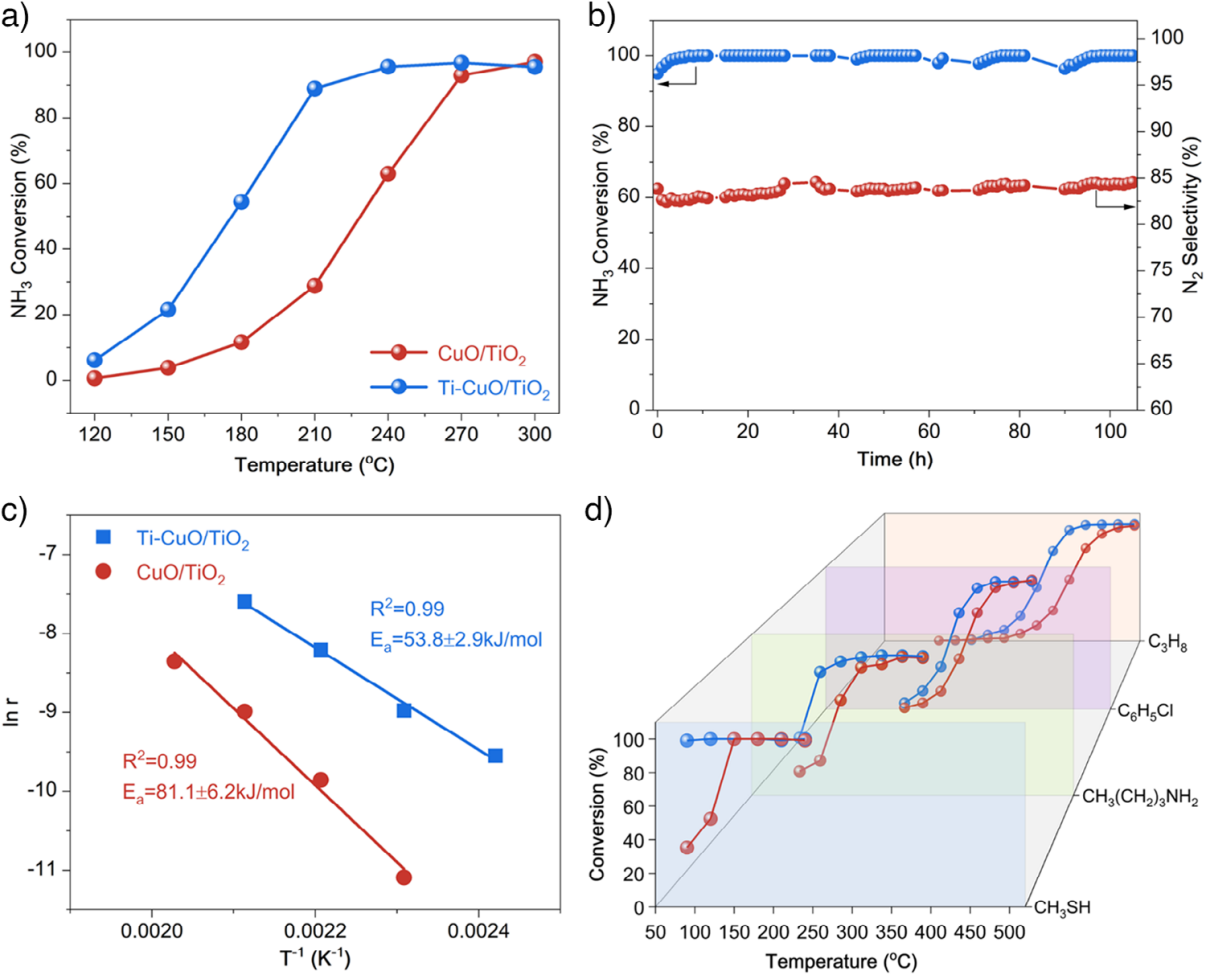
Catalytic oxidation performance. a) NH_3_ conversion as a function of temperature of Ti─CuO/TiO_2_ and CuO/TiO_2_. Conditions: [NH_3_] = 500 ppm, [O_2_] = 5 vol%, N_2_ as balance, and GHSV = 50 000 h^−1^. b) Long‐term NH_3_‐SCO performance of Ti─CuO/TiO_2_ and CuO/TiO_2_ catalysts. Conditions: T = 240 °C, [NH_3_] = 500 ppm, [O_2_] = 5 vol%, N_2_ as balance, and GHSV = 50 000 h^−1^. c) Arrhenius plot of NH_3_−SCO over Ti─CuO/TiO_2_ and CuO/TiO_2_. Conditions: [NH_3_] = 500 ppm, [O_2_] = 5 vol%, N_2_ as balance, and GHSV = 400 000 h^−1^. d) Heterogeneous catalytic oxidation performance of Ti─CuO/TiO_2_ (blue) and CuO/TiO_2_ (red) for CH_3_SH, CH_3_(CH_2_)_3_NH_2_, C_6_H_5_Cl, and C_3_H_8_. Conditions: [CH_3_SH] = 100 ppm, [O_2_] = 5 vol%, N_2_ as balance, and GHSV = 25 000 h^−1^ for CH_3_SH oxidation. [*n*‐butylamine] = 200 ppm, [O_2_] = 5 vol%, N_2_ as balance, and WHSV = 50 000 ml·g^−1^·h^−1^ for *n*‐butylamine oxidation. [C_6_H_5_Cl] = 100 ppm, [O_2_] = 10 vol%, N_2_ as balance, and GHSV = 50 000 h^−1^ for C_6_H_5_Cl oxidation. [C_3_H_8_] = 4000 ppm, [O_2_] = 5 vol%, Ar as balance, and WHSV = 100000 ml·g^−1^·h^−1^ for C_3_H_8_ oxidation.

The NH_3_‐SCO performance of Ti─CuO/TiO_2_ catalysts with different CuO loading amounts was evaluated (Figure S12). As seen in Figure 2a, the Ti─CuO/TiO_2_ catalyst with the optimum 10 wt% CuO loading (Table S5) shows 90% NH_3_ conversion at 210 °C, which is 60 °C lower than that of CuO/TiO_2_. The exceptional catalytic activity of Ti─CuO/TiO_2_ not only surpasses most reported Cu, Fe, and Mn‐based non‐precious metal catalysts but also rivals Pt, Pd, and Ag‐based precious metal catalysts (Table S6). The N_2_ selectivity of Ti─CuO/TiO_2_ could retain more than 80% below 270 °C (Figure S13). Furthermore, Ti─CuO/TiO_2_ exhibits excellent long‐term stability, with NH_3_ conversion maintained at nearly 100% for 105 h (Figure 2b). The used Ti─CuO/TiO_2_ catalyst maintains a highly dispersed state of CuO based on the XRD and HAADF‐STEM‐EDS analysis (Figures S14 and S15). No large decrease in activity during three consecutive recycling tests also demonstrates the good thermal stability of Ti─CuO/TiO_2_ (Figure S16). Some comparison CuO catalysts were also prepared using various styles of supports, including TiO_2_ with different crystalline phases (Figure S17), H_2_SO_4_ or H_3_PO_4_ treated TiO_2_ (Figure S18), as well as HNO_3_ treated CeO_2_ and Al_2_O_3_ (Figure S19). However, all these catalysts show inferior NH_3_‐SCO performance compared to Ti─CuO/TiO_2_. To investigate the intrinsic activity of catalysts, the reaction rates of catalysts were measured below 20% NH_3_ conversion. Ti─CuO/TiO_2_ shows over a 5‐fold increase in NH_3_ oxidation reaction rate compared with CuO/TiO_2_ at 180 °C (Figure S20). The Arrhenius plots and the apparent activation energy (*E*_a_) show that Ti─CuO/TiO_2_ has a much lower *E*_a_ (53.8 kJ mol^−1^) than that (81.1 kJ mol^−1^) of CuO/TiO_2_ (Figure 2c). In addition, Ti─CuO/TiO_2_ also shows higher turnover frequency (TOF) than that of CuO/TiO_2_ at temperatures of 150 °C, 180 °C, and 210 °C (Figure S21). These results suggest an enhanced intrinsic activity of Ti─CuO/TiO_2_ catalyst compared to CuO/TiO_2_. In addition to NH_3_ oxidation, the catalytic oxidation performance of various VOCs was also tested over Ti─CuO/TiO_2_ and CuO/TiO_2_. As shown in Figure 2d, Ti─CuO/TiO_2_ exhibits higher low‐temperature activity than CuO/TiO_2_ for the catalytic oxidation of CH_3_SH, CH_3_(CH_2_)_3_NH_2_, C_6_H_5_Cl, and C_3_H_8_. These results sufficiently prove that the Ti─CuO/TiO_2_ catalyst with Cu─O─Ti/Cu─O─Cu dual sites can be competent in various catalytic oxidation reactions.

**Figure 3:**
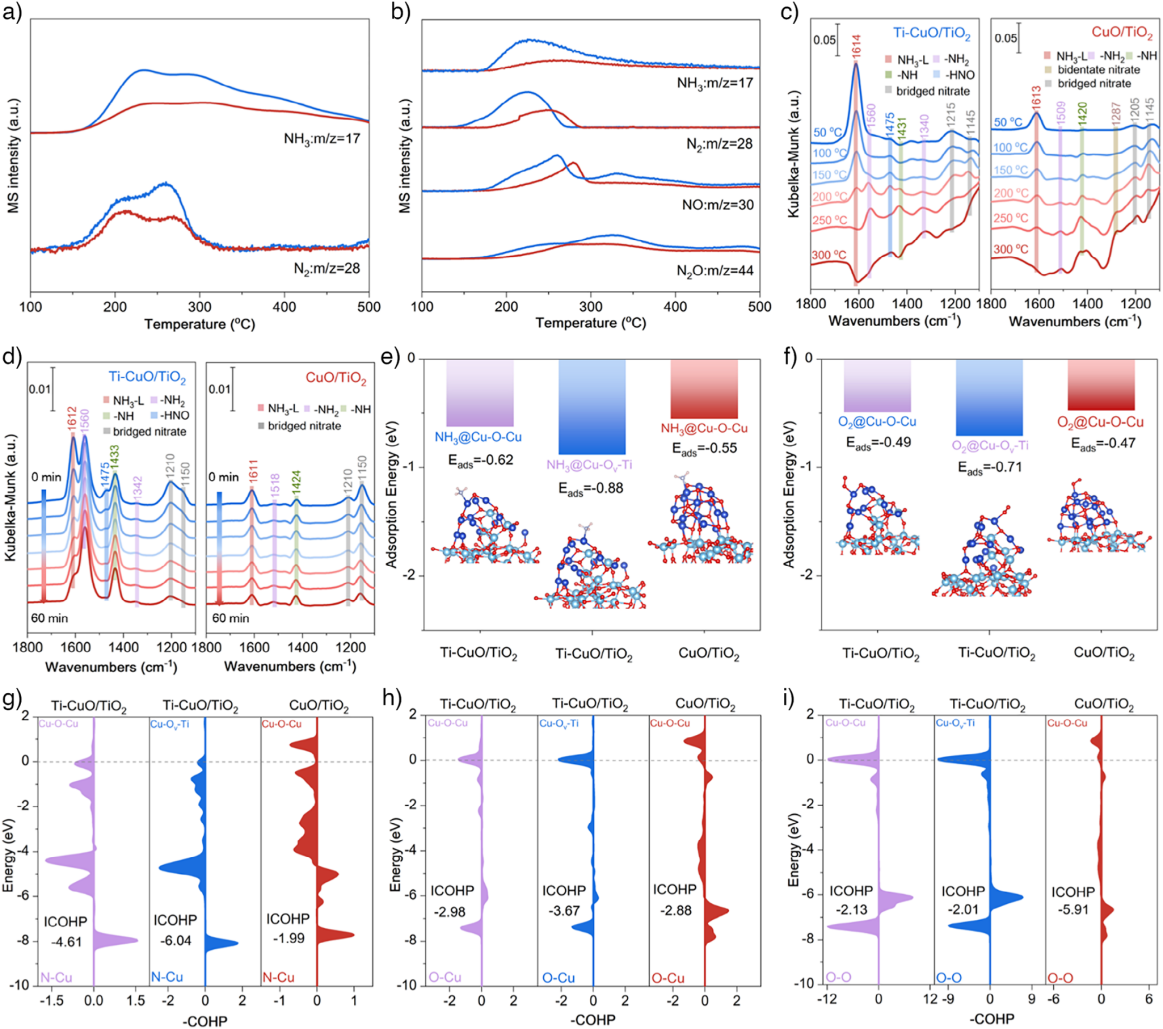
Adsorption and activation of NH_3_ and O_2_. a) MS signals of NH_3_ and N_2_ in NH_3_‐TPD. b) MS signals of NH_3_, N_2_, NO, and N_2_O in NH_3_‐O_2_‐TPSR. Catalysts: CuO/TiO_2_ (pink) and Ti─CuO/TiO_2_ (blue). c) In situ DRIFTS of NH_3_ adsorption of Ti─CuO/TiO_2_ and CuO/TiO_2_. d) In situ DRIFTS of the transient reaction between O_2_ and pre‐adsorbed NH_3_ at 200 °C of Ti─CuO/TiO_2_ and CuO/TiO_2_. Adsorption energy of NH_3_ e) and O_2_ f) on Cu─O─Cu/Cu─O_v_─Ti sites of Ti─CuO/TiO_2_ and Cu─O─Cu sites of CuO/TiO_2_. The COHP analysis of N─Cu bond g) and O─Cu bond h) for NH_3_ and O_2_ adsorption on Cu─O─Cu/Cu─O_v_─Ti sites of Ti─CuO/TiO_2_ and Cu─O─Cu sites of CuO/TiO_2_. i) The COHP analysis of O─O for O_2_ adsorption on Cu─O─Cu/Cu─O_v_─Ti sites of Ti─CuO/TiO_2_ and Cu─O─Cu sites of CuO/TiO_2_. All the Fermi levels (*E*_F_) are set to 0 eV.

As there are no large variations in textural properties between the two catalysts (Table S7), the textural properties of catalysts are unlikely to be the primary factors driving the significant activity differences. In order to probe the nature of the reason for the excellent intrinsic activity of Ti─CuO/TiO_2_, the adsorption and activation behaviors of the NH_3_ and O_2_ reactants were investigated. NH_3_ temperature‐programmed desorption (NH_3_‐TPD) shows that Ti─CuO/TiO_2_ could adsorb more NH_3_ than CuO/TiO_2_ (Figure 3a), demonstrating an enhanced NH_3_ adsorption capacity of Cu─O─Ti/Cu─O─Cu dual sites. During NH_3_‐TPD, N_2_ generation resulted from the NH_3_ oxidation by surface or bulk lattice oxygen species on the catalysts. Clearly, more N_2_ production is noticed on Ti─CuO/TiO_2_, suggesting more reactive oxygen species and higher oxygen mobility over Cu─O─Ti/Cu─O─Cu dual sites, which is also evidenced by the O 1s XPS spectra and O_2_‐TPD results (Figures S22 and S23). The adsorption and activation capacities of NH_3_ and O_2_ were further probed by temperature‐programmed surface reaction (TPSR) conducted by introducing O_2_ after the pre‐adsorption of NH_3_ (Figure 3b). During the TPSR, more N_2_ formation at a lower temperature is achieved on Ti─CuO/TiO_2_ compared to CuO/TiO_2_. Besides, NO and N_2_O as NH_3_ oxidation products could also generate at a lower temperature over Ti─CuO/TiO_2_ than CuO/TiO_2_. These results also demonstrate the stronger oxidative capacity over Ti─CuO/TiO_2_. In situ diffuse reflectance infrared Fourier transform spectroscopy (DRIFTS) of NH_3_ adsorption of two catalysts demonstrates a stronger NH_3_ adsorption ability of Ti─CuO/TiO_2_ (Figure 3c). Compared with CuO/TiO_2_, in addition to the adsorbed NH_x_ species, the HNO species (∼1475 cm^−1^)^[^ ^21^ ^]^ can be found on Ti─CuO/TiO_2_, which is the intermediate of the imide reaction pathway. In situ DRIFTS of the transient reaction between O_2_ and pre‐adsorbed NH_3_ at 200 °C (Figure 3d) demonstrate an obvious decrease of adsorbed NH_3_ species along with the formation of NH_x_ species after introducing O_2_ over Ti─CuO/TiO_2_ but no changes occur on CuO/TiO_2_. Interestingly, it can be found that the decrease of HNO species after introducing O_2_, implies the high reactivity of HNO with NH to generate N_2_ and H_2_O via the imide mechanism. Based on the above results, it can be concluded that Ti─CuO/TiO_2_ owns a stronger oxygen activation ability that boosts the NH_3_ oxidation to N_2_ via the imide reaction pathway between HNO and NH intermediates. As seen in Figure 3e, the NH_3_ adsorption energy is −0.88 and −0.62 eV for the NH_3_ adsorbed on Cu─O_v_─Ti and Cu─O─Cu sites of Ti─CuO/TiO_2_, while it is −0.55 eV on Cu─O─Cu sites of CuO/TiO_2_. Clearly, the Cu─O_v_─Ti sites of Ti─CuO/TiO_2_ are more favorable for NH_3_ adsorption. For the adsorption of O_2_ (Figure 3f), the O_2_ adsorption energy is −0.71 and −0.49 eV for the O_2_ adsorbed on Cu─O_v_─Ti and Cu─O─Cu sites of Ti─CuO/TiO_2_, while it is −0.47 eV on Cu─O─Cu sites of CuO/TiO_2_. Notably, the Cu─O_v_─Ti sites of Ti─CuO/TiO_2_ are more favorable for the adsorption of O_2_. Furthermore, crystal orbital Hamilton populations (COHPs) were also performed to investigate the adsorption capacity of NH_3_ and O_2_ on Cu─O_v_─Ti/Cu─O─Cu sites of Ti─CuO/TiO_2_ and Cu─O─Cu sites in CuO/TiO_2_. The more negative value of the integrated COHP (ICOHP) up to the Fermi level (*E*_F_) indicates a stronger bonding interaction. The ICOHP value of the Cu─N bond is −4.61 and −6.04 on Cu─O─Cu and Cu─O_v_─Ti sites of Ti─CuO/TiO_2_ and −1.99 on Cu─O─Cu sites in CuO/TiO_2_ (Figure 3g), which indicates that the Cu─O_v_─Ti sites of Ti─CuO/TiO_2_ have the highest bonding state of NH_3_. The ICOHP value of the Cu─O/O─O bond is −2.98/−2.13 and −3.67/−2.01 on Cu─O─Cu and Cu─O_v_─Ti sites of Ti─CuO/TiO_2_ and −2.88/−5.91 on Cu─O─Cu sites in CuO/TiO_2_, respectively (Figure 3h,i). This result evidences that Cu─O_v_─Ti sites of Ti─CuO/TiO_2_ own the strongest O_2_ adsorption and activation capacity due to the high bonding state of O_2_ on Cu─O_v_─Ti sites and weak O─O bonding strength. The existence of Cu─O_v_─Ti sites contributes to the O_2_ activation and formation of HNO species at 200 °C (Figure 3d), similar to the Pt,^[^ ^22^ ^]^ Ag,^[^ ^23^ ^]^ and Au^[^ ^24^ ^]^‐based precious metal catalysts. Therefore, the imide reaction pathway can proceed at low temperatures between HNO and NH species adsorbed on Cu─O_v_─Ti and Cu─O─Cu sites of Ti─CuO/TiO_2_ respectively because of the enhanced adsorption and activation capacity of NH_3_ and O_2_. However, the weaker O_2_ activation capacity of CuO/TiO_2_ only generates NH_x_ species without nitrogen oxide species at low temperatures, which impedes the progress of the i‐SCR reaction pathway (Figure 3d).

**Figure 4:**
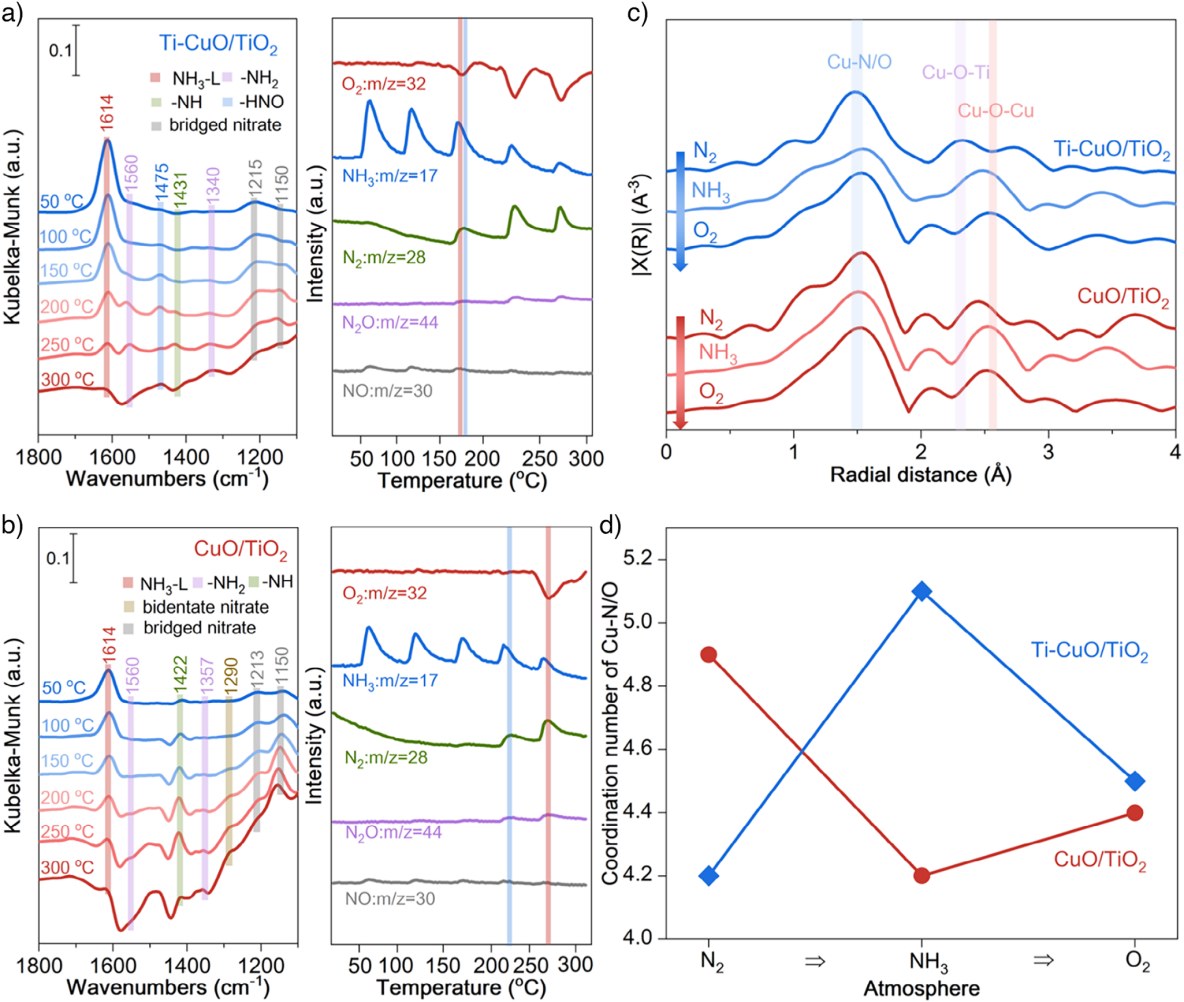
Mechanism investigation. a) In situ DRIFTS and corresponding MS signals recorded during NH_3_ oxidation of Ti─CuO/TiO_2_. b) In situ DRIFTS and corresponding MS signals recorded during NH_3_ oxidation of CuO/TiO_2_. c) In situ EXAFS spectra of the transient reaction between O_2_ and pre‐adsorbed NH_3_ at 200 °C of Ti─CuO/TiO_2_ and CuO/TiO_2._ d) Corresponding coordination number of Cu─N/O.

Furthermore, the intermediates and pathways of the SCO reaction were explored by in situ DRIFTS with MS monitoring, as shown in Figure 4a,b. As for Ti─CuO/TiO_2_, the NH_3_ species adsorbed on Lewis acid sites are observed after introducing NH_3_ and O_2_ at 50 °C for 1 h.^[^ ^25^ ^]^ With the increase of temperature, the NH_3_ species gradually decrease, while the ─NH_2_ (1560 and 1340 cm^−1^),^[^ ^26^ ^]^ ─NH (1431 cm^−1^),^[^ ^27^ ^]^ HNO (1475 cm^−1^)^[^ ^21^ ^]^ and bridged nitrate species (1215 and 1150 cm^−1^)^[^ ^28^ ^]^ present a trend of increase and then decrease. The corresponding MS signals exhibited the N_2_ production alongside O_2_ consumption at 150 °C. The much weaker N_2_O and NO_x_ signal also indicates the good N_2_ selectivity over Ti─CuO/TiO_2_. For CuO/TiO_2_, with the increase of temperature, the adsorbed NH_3_ species gradually decrease while NH_2_ (1560 and 1357 cm^−1^)^[^ ^26^ ^]^ ─NH (1422 cm^−1^)^[^ ^27^ ^]^ and bridged nitrate species (1213 and 1150 cm^−1^)^[^ ^28^ ^]^ species also present a trend of increase and then decrease. Whereas the bidentate nitrate species (1290 cm^−1^)^[^ ^29^ ^]^ gradually increase with increasing temperature, indicating the inferior reactivity. The corresponding MS signals exhibited the N_2_ production and O_2_ consumption at higher temperatures of 200 °C and 250 °C, respectively. These results prove that Ti─CuO/TiO_2_ has a stronger oxygen activation capacity that boosts the rapid reaction between ─NH and ─HNO through the imide reaction pathway below 200 °C. Both catalysts also can conduct the reaction between adsorbed NH_x_ and NO_x_ through i‐SCR above 200 °C.

In situ EXAFS and XANES of the transient reaction between O_2_ and pre‐adsorbed NH_3_ at 200 °C were carried out to show the alteration of the coordination environment and valence state of Cu. As for Ti─CuO/TiO_2_ (blue), the Cu─O─Ti (second shell) vanishes after 1 h of NH_3_ exposure, coinciding with an increase from 4.2 to 5.1 in the coordination number of Cu─O/N bonds (Figure 4c,d and Table S8). This result implies that the Cu─O─Ti sites can coordinate with NH_3_ to form Cu─N bonds, which also increases the Cu valence state due to the higher energy of the Cu‐K XANES edge (Figure S24).^[^ ^30^ ^]^ Interestingly, after introducing O_2_ for 1 h, the peak ascribed to Cu─O─Ti (second shell) reappears, and the coordination number of the Cu─O/N bond decreases to 4.5 (Figure 4c,d and Table S8), which reveals that the coordinated NH_3_ on Cu─O─Ti sites is consumed due to the reaction with oxygen species. Correspondingly, the Cu valence state also decreases due to the depletion of Cu─N bonds (Figure S24). For CuO/TiO_2_ (pink), the coordination number of Cu─O/N initially decreases after exposure to NH_3_ for 1 h because of the lattice oxygen depletion during NH_3_ activation, which also decreases the Cu valence due to the decreased energy of the Cu─K XANES edge (Figures 4c,d, and S25; Table S9). Whereas, upon oxygen introduction, the coordination number of Cu─O/N does not recover significantly, and the energy of the Cu‐K XANES edge is also almost unchanged, indicating that lattice oxygen replenishment is hindered by inadequate O_2_ activation (Figures 4c,d, and S25; Table S9).

**Figure 5:**
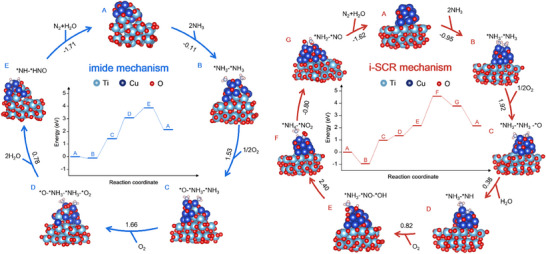
Proposed mechanisms of NH_3_ oxidation on Ti─CuO/TiO_2_ (blue) and CuO/TiO_2_ (pink).

The imide and i‐SCR reaction pathways were investigated on Ti─CuO/TiO_2_ and CuO/TiO_2_ surfaces by DFT calculations, respectively (Figure 5). As for Ti─CuO/TiO_2_, two key *HNO and *NH intermediates of the imide mechanism form on neighboring Cu─O─Ti and Cu─O─Cu sites, respectively, as detailed in Figure 5 (blue). First, two NH_3_ molecules adsorb on Cu─O─Ti and Cu─O─Cu sites of Ti─CuO/TiO_2_ with an exothermic −0.11 eV. Then, 1/2 O_2_ and O_2_ adsorb on oxygen vacancy (O_V_) of CuO_x_ and Cu─O─Ti with endothermic 1.53 and 1.66 eV, respectively. Subsequently, the neighboring *NH_3_ and *O_2_ on Cu─O─Ti sites transform to *HNO intermediates and H_2_O while the neighboring *NH_3_ and *O on CuO_x_ sites transform to *NH intermediates and H_2_O, with a totally an endothermic 0.78 eV. Lastly, an N─N coupling process occurs between the neighboring *NH and *HNO intermediates to form N_2_ and H_2_O with an exothermic −1.71 eV, leading to a clean Ti─CuO/TiO_2_ surface. Besides, we also considered the possibility of *NH oxidation to *NO, which is the key intermediate of the i‐SCR mechanism. Whereas, *NH reacts with *O to form *NO along with *H transfer onto adjacent *O to form *OH, which is endothermic at 1.03 eV (Figure S26). Therefore, the direct reaction between *NH and *HNO to form N_2_ and H_2_O is more preferred. From the reaction free energy profile (inset of Figure 5, blue line), the *O adsorption on O_V_ of Cu─O─Ti requires the most energy, 1.66 eV, which is supposed to be the rate‐limiting step of the imide reaction pathway. As for CuO/TiO_2_, the i‐SCR reaction pathway happens as illustrated in Figure 5 (pink). Two NH_3_ molecules adsorb on CuO_x_ sites with an exothermic −0.95 eV, following the adsorption of 1/2 O_2_ in O_V_ with endothermic 1.92 eV. The oxidation dehydrogenation of *NH_3_ generates *NH species and H_2_O with an endothermic 0.38 eV. Then, NH reacts with *O to form *NO along with *H transfer onto adjacent *O to form ─OH, which is endothermic at 0.82 eV. Subsequently, *NO─*OH─*NH_3_ transforms to the SCR intermediate *NO_2_─*NH_4_ with endothermic 2.40 eV. And *NO_2_─*NH_4_ further transforms to *NO─*NH_2_ and a H_2_O with an exothermic −0.80 eV. Lastly, *NO─*NH_2_ decomposes to N_2_ and H_2_O with an exothermic −1.62 eV, leading to a clean CuO/TiO_2_ surface. From the reaction free energy profile (inset of Figure 5, pink line), the formation of *NO_2_─*NH_4_ intermediates requires the most energy, 2.40 eV, which is supposed to be the rate‐limiting step of the i‐SCR reaction pathway. From the results of DFT results, the imide reaction pathway over Ti─CuO/TiO_2_ requires lower reaction barriers than i‐SCR over CuO/TiO_2_, indicating that the SCO reaction is more thermodynamically favorable over Ti─CuO/TiO_2_ with gradient oxidative Cu─O─Ti/Cu─O─Cu dual sites.

## Conclusion

In this study, we designed and successfully synthesized a TiO_2_ supported Ti dopped CuO catalyst, which exhibits superior catalytic oxidation activity of NH_3_ and various VOCs at low temperatures compared to other reported catalysts for these reactions. Comprehensive in situ characterizations combined with DFT calculations demonstrate that the gradient oxidative Cu─O─Ti/Cu─O─Cu dual sites in this catalyst play crucial roles in low‐temperature catalytic oxidation of NH_3_. The Cu─O─Ti sites—with stronger oxidative capacity—contribute to the activation of O_2_ and NH_3_, thus promoting the formation of HNO intermediates. The adjacent Cu─O─Cu sites—with weaker oxidative capacity—facilitate the formation of NH intermediates. The HNO intermediates react with the NH intermediates, generating N_2_ and H_2_O at low temperature via an imide mechanism, thus effectively lowering the reaction barrier for the NH_3_ catalytic oxidation. As such, this work pioneers a novel strategy for boosting catalytic oxidation reactions by engineering highly active dual sites to optimize bimolecular activation and to open new reaction pathways in oxidation reactions.

## Supporting Information

The detailed experimental section, additional figures tables are listed in the Supporting Information file. The authors have cited additional references within the Supporting Information.

## Conflict of Interests

E.C. is an International Consultant of the Academic Committee of the Carbon Neutrality Research Center at Shanghai University, China.

## Supporting information



Supplementary Information

## Data Availability

The data that support the findings of this study are available from the corresponding author upon reasonable request.
