# Supplementary material for: Non‐Precious Metal Catalysts with Gradient Oxidative Dual Sites Boost Bimolecular Activation for Catalytic Oxidation Reactions
Source: Angew Chem Int Ed Engl. 2025 Apr 21;64(25):e202506018. doi: 10.1002/anie.202506018 (PMC12171384; doi:10.1002/anie.202506018)
Supplement: Supplementary file 1 — Supplementary Information [file ANIE-64-e202506018-s001.docx]

***Supporting Information***

**Non-Precious Metal Catalysts with Gradient Oxidative Dual Sites Boost Bimolecular Activation for Catalytic Oxidation Reactions**

Yufei Wang,^[a]^ Tianwei Lan,^[a]^ Lupeng Han,*^[a]^ Evangelina Pensa,^[b]^ Yongjie Shen,^[c]^ Xingchi Li,^[a]^ Zixiang Xu,^[a]^ Xin Chen,^[a]^ Mengxue Wang,^[a]^ Xiaoya Xue,^[a]^ Yanqing Li,^[a]^ Ming Xie,^[d]^ Emiliano Cortés,*^[b]^ Dengsong Zhang*^[a]^

[a] Y. Wang, T. Lan, Assoc.Prof. L. Han, X. Li, Z. Xu, X. Chen, M. Wang, X. Xue, , Y. Li, Prof. D. Zhang
International Joint Laboratory of Catalytic Chemistry, State Key Laboratory of Advanced Special Steel, Innovation Institute of Carbon Neutrality, Department of Chemistry, College of Sciences, Shanghai University, Shanghai 200444, People’s Republic of China
E-mail: [lphan@shu.edu.cn](mailto:lphan@shu.edu.cn) and [dszhang@shu.edu.cn](mailto:dszhang@shu.edu.cn)

[b] E.Pensa, Prof. E. Cortés
Nanoinstitute Munich, Faculty of Physics, Ludwig-Maximilians-Universität (LMU), Munich 80539, Germany.
E-mail: [Emiliano.Cortes@lmu.de](mailto:Emiliano.Cortes@lmu.de)

[c] Y. Shen
Institute for Chemical Reaction Design and Discovery (WPI-ICReDD), Hokkaido University, Sapporo 001-0021, Japan.

[d] M. Xie
Department of Chemical Engineering, University of Bath, BA27AY, Bath, UK.

**Contents**

1. Materials and Reagents
2. Experimental Section
3. Figures and Tables

Figure S1. Schematic illustration of the preparation method.

Figure S2. N 1s XPS spectrum of TiO_2_-NA and TiO_2_.

Figure S3. FT-IR spectra of TiO_2_-NA and TiO_2_.

Figure S4. XRD patterns of Ti-CuO/TiO_2_ and CuO/TiO_2_.

Figure S5. HAADF-STEM images with the corresponding EDS mappings for Ti-CuO/TiO_2_.

Figure S6. HAADF-STEM images with the corresponding EDS mappings for CuO/TiO_2_.

Figure S7. HRTEM images for CuO/TiO_2_.

Figure S8. HRTEM images for Ti-CuO/TiO_2_.

Figure S9. Optimized model for Ti-CuO/TiO_2_ catalyst.

Figure S10. Optimized model for CuO/TiO_2_ catalyst.

Figure S11. Bond length of Cu-O in Ti-O-Cu-O-Cu-O-Cu structure.

Figure S12. NH_3_ conversion (a) and N_2_ selectivity (b) as a function of temperature of Ti-CuO/TiO_2_ catalysts with the CuO loading of 5 wt%, 10 wt%, and 15 wt%.

Figure S13. N_2_ selectivity as a function of temperature of Ti-CuO/TiO_2_ and CuO/TiO_2_.

Figure S14. XRD patterns of Ti-CuO/TiO_2_ and Ti-CuO/TiO_2_ after the durability test.

Figure S15. HAADF-STEM images with the corresponding EDS mappings for Ti-CuO/TiO_2_ after the durability test.

Figure S16. The cycling NH_3_-SCO performance. NH_3_ conversion (a) and N_2_ selectivity (b) of Ti-CuO/TiO_2_ catalyst.

Figure S17. NH_3_ conversion and N_2_ selectivity as a function of temperature of Ti-CuO/TiO_2_, CuO/TiO_2_-A, CuO/TiO_2_-R and CuO/TiO_2_-P25.

Figure S18. NH_3_ conversion as a function of temperature of Ti-CuO/TiO_2_, CuO/TiO_2_-P and CuO/TiO_2_-S.

Figure S19. NH_3_ conversion and N_2_ selectivity as a function of temperature of Ti-CuO/TiO_2_, CuO/Al_2_O_3_ and CuO/ Al_2_O_3_-N (a) and Ti-CuO/TiO_2_, CuO/CeO_2_ and CuO/ CeO_2_-N (b).

Figure S20. Reaction rates at 180 °C of Ti-CuO/TiO_2_ and CuO/TiO_2_.

Figure S21. TOF of Ti-CuO/TiO_2_ and CuO/TiO_2_ at temperatures of 150°C, 180°C, and 210°C.

Figure S22. O 1s XPS spectra of Ti-CuO/TiO_2_ and CuO/TiO_2_.

Figure S23. MS signals in O_2_-TPD of Ti-CuO/TiO_2_ and CuO/TiO_2_.

Figure S24. *In situ* Cu K-edge XANES spectra of the transient reaction between O_2_ and pre-adsorbed NH_3_ at 200 ^o^C of Ti-CuO/TiO_2_.

Figure S25. *In situ* Cu K-edge XANES spectra of the transient reaction between O_2_ and pre-adsorbed NH_3_ at 200 ^o^C of CuO/TiO_2_.

Figure S26. Calculated energy of *NH before and after reacting with *O (pink) and *HNO (blue) on Ti-CuO/TiO_2_, respectively.

Table S1. The result of dissociative N_2_O oxidation and H_2_ titration for Ti-CuO/TiO_2_ and CuO/TiO_2_.

Table S2. Relative area proportion of Cu^2+^ and Cu^+^ in XPS for Ti-CuO/TiO_2_ and CuO/TiO_2_.

Table S3. Fit parameters of EXAFS data of Ti-CuO/TiO_2_ catalyst.

Table S4. Fit parameters of EXAFS data of CuO/TiO_2_ catalyst.

Table S5. Copper and CuO contents in Ti-CuO/TiO_2_ from ICP-OES.

Table S6. Comparison upon NH_3_-SCO performance of the catalyst in this work with the reported catalysts.

Table S7. Specific surface area (S_BET_) and pore properties of Ti-CuO/TiO_2_ and CuO/TiO_2_.

Table S8. Fit parameters of *in-situ* EXAFS data of the transient reaction between O_2_ and pre-adsorbed NH_3_ at 200 ^o^C of Ti-CuO/TiO_2_.

Table S9. Fit parameters of *in-situ* EXAFS data of the transient reaction between O_2_ and pre-adsorbed NH_3_ at 200 ^o^C of CuO/TiO_2_.

1. Materials and Reagents

All of the chemicals were of analytical reagent grade and used as received without any further purification.

2. Experimental Section

**Synthesis of supports.**

The TiO_2_-NA was synthesized via a HNO_3_ modified sol-gel method. Firstly, 9 mL of tetrabutyl titanate was first dispersed into 66 mL of absolute ethanol. Then this solution was added into 90 mL of deionized water dropwise under vigorous stirring. The resulting suspension was then heated to 70 °C for 2 h under mechanical stirring to evaporate most of the solvent. Next, 300 mL of 0.3 M NaOH aqueous solution was added to the suspension. After continuous stirring for another 12 h, the precipitate was washed with deionized water several times to obtain hydroxy-rich layered titanate. In the next nitrate group grafting section, 12.5 g of this wet titanate was directly mixed with 120 mL 1M dilute HNO_3_ in a 200 mL flask and then refluxed at 80 °C for 4 h under continuous stirring. The final precipitate was collected via centrifugation and washed with ethanol and deionized water, then dried in a vacuum oven at 80 °C for 12 h. The TiO_2_ was obtained by calcination of TiO_2_-NA at 500 ℃ for 2 h (heating ramp of 5 °C·min^-1^). TiO_2_-P and TiO_2_-S were synthesized following the identical procedure as TiO_2_-NA, with the modification of replacing nitric acid with phosphoric acid and sulfuric acid, respectively. **Al_2_O_3_-N and CeO_2_-N were subjected to the same nitrate group grafting procedure as TiO_2_-NA, with the exception that wet titanate was replaced by 0.5 g commercial Al_2_O_3_ or CeO_2_, respectively.**

**Synthesis of CuO-based catalysts.**

The Ti-CuO/TiO_2_ and CuO/TiO_2_ catalysts were prepared by a facile impregnation of TiO_2_-NA and TiO_2_ with a certain amount of copper nitrate aqueous solutions according to 10 wt% CuO loading. Then, the samples were obtained after the evaporation of water at 60 °C and calcination at 500 °C for 2 h (heating ramp of 5 °C·min^-1^) in air. TiO_2_-P, TiO_2_-S, TiO_2_-A, TiO_2_-R, TiO_2_-P25, CeO_2_, CeO_2_-N, Al_2_O_3_ and Al_2_O_3_-N supported CuO catalysts were synthesized through an identical method as that of Ti-CuO/TiO_2_ except for different supports. TiO_2_-A, TiO_2_-R, TiO_2_-P25 are commercial TiO_2_ with anatase phase, rutile phase, and a mixed-phase of anatase and rutile, respectively.

**Structural characterization.**

X-ray diffraction (XRD) patterns were collected on a Bruker D8 Advance diffractometer equipped with a Cu Kα radiation source. N_2_ physical adsorption-desorption was processed on a Micro-Active ASAP 2460 analyzer instrument. The specific surface area was calculated based on the Brunauer-Emmett-Teller (BET) model and pore properties were calculated based on the Barret-Joyner-Halenda (BJH) model. High-resolution transmission electron microscopy (HRTEM), high angle annular dark field scanning transmission electron microscopy (HAADF-STEM) images and energy dispersive X-ray spectroscopy (EDS) mappings were collected by a JEOL JEM-F200 field-emission electron microscopy equipped with an EDS. Fourier Transform infrared spectroscopy (FT-IR) was collected on a Nicolet 6700 spectrometer equipped with a mercury-cadmium-telluride detector. Inductively coupled plasma-optical emission spectrometer (ICP-OES) was conducted on Agilent720ES. The surface chemical valence was studied by a X-ray photoelectron spectroscopy (XPS) system (PHI-5300) with Mg Kα radiation. X-ray Absorption Fine Structure (XAFS) spectra of Cu K-edge was carried out using the Rapid XAFS 2M (Anhui Absorption Spectroscopy Analysis Instrument Co. Ltd.) by transmission（or fluorescence）mode at 20 kV and 30 mA. And the Si (553) spherically bent crystal analyzer with a radius of curvature of 500 mm was used. For *in situ* investigations, 40 mg of the catalyst was diluted with 60 mg B_3_N_4_ and pressed to a slice. Gases were supplied via mass flow controllers and the total gas flow was set to 30 ml/min. The catalyst was pre-treated in N_2_ for 30min at 300 ^o^C and then reduced to 200 ^o^C. After that, 30 mL·min^-1^ gas flow containing 1000 ppm NH_3_ was continuously introduced for 60 min. And then, NH_3_ gas was shut off and 5% O_2_/He was injected for 60 min. The spectra were collected before introducing NH_3_, after introducing NH_3_ and after introducing O_2_, respectively.

**Catalytic measurements.**

Catalytic performance of all catalysts in NH_3_-SCO (20−40 mesh), CH_3_SH-SCO, n-butylamine-SCO, C_6_H_5_Cl-SCO and C_3_H_8_-SCO (40−60 mesh) was evaluated in a fixed bed reactor. For NH_3_-SCO, [NH_3_] = 500 ppm, [O_2_] = 5 vol%, N_2_ as balance and gas hourly space velocity (GHSV) = 50,000 h^-1^. For CH_3_SH-SCO, [CH_3_SH] = 100 ppm, [O_2_] = 5 vol%, N_2_ as balance and GHSV = 25,000 h^-1^. For n-butylamine-SCO, [n-butylamine] = 200 ppm, [O_2_] = 5 vol%, N_2_ as balance and WHSV = 50,000 ml·g^-1^·h^-1^. For C_6_H_5_Cl-SCO, [CB] = 100 ppm, [O_2_] = 10 vol%, N_2_ as balance and GHSV = 50,000 h^-1^. And for C_3_H_8_-SCO, [C_3_H_8_] = 4000 ppm, [O_2_] = 5 vol%, Ar as balance and WHSV = 100,000 ml·g^-1^·h^-1^. The composition of the tail gas was analyzed quantitatively by a Fourier transform infrared multicomponent gas analyzer (Thermo Scientific Antaris IGS Analyzer). Catalytic conversion (X) and selectivity (S) were calculated using the following equations:

(S1) $X_{{NH}_{3}}=\left( \frac{\left[ {NH}_{3_{inlet}} \right]-\left[ {NH}_{3_{outlet}} \right]}{\left[ {NH}_{3_{inlet}} \right]} \right)\times100\%$

(S2) $S_{N_{2}}=\left( \frac{\left[ {NH}_{3_{inlet}} \right]-\left[ {NH}_{3_{outlet}} \right]-\left[ {NO}_{outlet} \right]-\left[ {NO}_{2_{outlet}} \right]-2\times\left[ {N_{2}O}_{outlet} \right]}{\left[ {NH}_{3_{inlet}} \right]-\left[ {NH}_{3_{outlet}} \right]} \right)\times100\%$

(S3) $X_{CH_{3}SH}=\left( \frac{\left[ CH_{3}SH_{inlet} \right]+2\times\left[ CH_{3}SCH_{3_{inlet}} \right]-\left[ CH_{3}SH_{outlet} \right]-2\times\left[ CH_{3}SCH_{3_{outlet}} \right]}{\left[ CH_{3}SH_{inlet} \right]+2\times\left[ CH_{3}SCH_{3_{inlet}} \right]} \right)\times100\%$

(S4) $X_{n-\mathrm{butylamine}}=\left( \frac{\left[ n-\mathrm{butylamin}e_{inlet} \right]-\left[ n-\mathrm{butylamin}e_{outlet} \right]}{\left[ n-\mathrm{butylamin}e_{inlet} \right]} \right)\times100\%$

(S5) $X_{C_{6}H_{5}Cl}=\left( \frac{\left[ C_{6}H_{5}{Cl}_{inlet} \right]-\left[ C_{6}H_{5}{Cl}_{outlet} \right]}{\left[ C_{6}H_{5}{Cl}_{inlet} \right]} \right)\times100\%$

(S6) $X_{C_{3}H_{8}}=\left( \frac{\left[ C_{3}H_{8_{inlet}} \right]-\left[ C_{3}H_{8_{outlet}} \right]}{\left[ C_{3}H_{8_{inlet}} \right]} \right)\times100\%$

Kinetic experiments were conducted by the same procedure with NH_3_ conversion restrained under 20% by varying the weight of catalyst. The apparent activation energies (E_a_ in kJ/mol) over the catalysts were determined following the Arrhenius equation.

Turnover frequency (TOF) was calculated according to formula S7:

(S7) $TOF(s^{-1})=\frac{\eta_{(NH_{3})}*Q/(60*V_{m})}{(m*\omega_{Cu}*D_{Cu})/M_{Cu}}$

Where η_(NH3)_, Q, V_m_, m, M_Cu_, and ω_Cu_ represent the NH_3_ conversion (%), the total flow rate (L⋅min^-1^), molar volume of gas (22.4 L⋅mol^-1^), the catalyst weight (g), copper molar mass (g⋅mol^-1^), and copper loading (%), respectively. D_Cu_ is Cu dispersion calculated according to the result of dissociative N_2_O oxidation and H_2_ titration.

**Chemisorption analysis.**

Temperature-programmed experiments including NH_3_-TPD, O_2_-TPD, and NH_3_-O_2_-TPSR were operated on a Micrometrics Auto Chem 2920 instrument coupled with a MS (Pfeiffer Vacuum GSD 320). In NH_3_-TPD, all samples (80 mg) were pretreated in He at 300 °C in a quartz reactor for 30 min, and then 10% NH_3_/He gas flow was introduced at 100 °C for 60 min for the NH_3_ adsorption. After that, He was introduced to purge the physiosorbed NH_3_ and stabled the baseline. Chemisorption profiles and MS signals were collected from 100 °C to 500 °C. In O_2_-TPD, all samples (80 mg) were pretreated in He at 300 °C for 30 min and then 2% O_2_/He was introduced at 100 °C for 60 min. After introducing He for another 60 min to purge impurities and stable the baseline, chemisorption profiles and MS signals were collected from 100 °C to 900 °C. In NH_3_-O_2_-TPSR, all catalysts (80 mg) were pretreated in He at 300 °C for 30 min, and 30 mL·min^-1^ 10% NH_3_/He gas flow was introduced for NH_3_ adsorption at 100 ^o^C. After being purged by He for 30 min, 30 mL·min^-1^ 2% O_2_/He gas flow was introduced and MS signals were recorded from 100 °C to 500 °C. The dispersion of copper (D_Cu_) was measured by dissociative N_2_O oxidation and H_2_ titration. Typically, 50 mg catalysts were first pretreated in a helium flow at 200 ^o^C for 1 h and cooled to room temperature. Then catalysts were reduced in a 10% H_2_/Ar mixture from room temperature to 300 ^o^C for 2 h. The hydrogen consumption from this reduction process was assigned as A_1_. Pure He (30 mL/min) was purged to the sample until 50 ^o^C. The catalyst was oxidized by 10 % N_2_O/He (30 mL/min) at 90 ^o^C for 1.5 h. Subsequently, pure He (30 mL/min) flowed through the sample for 0.5 h to remove the residual N_2_O. Finally, another reduction process was performed in 10% H_2_/Ar (30 mL/min), and the hydrogen consumption was denoted as A_2_. The D_Cu_ was calculated by the following equation of D_Cu_ = 2A_2_/A_1_. Diffuse reflectance infrared Fourier transform spectroscopy (DRIFTS) measurements were collected on a VERTEX 80v spectrometer equipped with a mercury-cadmium-telluride detector coupled with a MS (Hiden DECRA). As for *in situ* DRIFTS recorded during NH_3_ adsorption, all samples were pre-treated in 30 mL·min^-1^ He at 300 °C for 30 min and background files were collected at different temperatures. After that, 1000 ppm NH_3_ in 30 mL·min^-1^ NH_3_/He gas was continuously introduced at 50 °C for 60 min, and then spectra were collected at different temperatures. For *in situ* DRIFTS recorded during the transient reaction of passing O_2_ after preabsorbed with NH_3_, all samples were pre-treated in 30 mL·min^-1^ He at 300 °C for 30 min and background files were collected at 200 ^o^C. After that, 30 mL·min^-1^ gas flow containing 1000 ppm NH_3_ was continuously introduced for 60 min. And then, NH_3_ gas was shut off and 5% O_2_/He was injected for 60 min while collecting spectra. For *in situ* DRIFTS recorded during NH_3_ oxidation, all samples were pre-treated in 30 mL·min^-1^ He at 300 °C for 30 min and background files were collected at different temperatures. After that, 30 mL·min^-1^ gas flow containing 1000 ppm NH_3_ and 5% O_2_ is continuously introduced into the reactor at 50 °C for 60 min, and spectra at different temperature were collected accompanied with MS monitoring.

**Density functional theory (DFT) calculations.**

Density functional theory (DFT) implemented in the Vienna ab initio simulation package (VASP) was used to perform calculations. The exchange-correlation potential is described by using the generalized gradient approximation of Perdew-Burke-Ernzerhof (GGA-PBE). The projector augmented-wave (PAW) method is employed to treat interactions between ion cores and valence electrons. The plane-wave cutoff energy was fixed to 450 eV. Given structural models were relaxed until the Hellmann–Feynman forces smaller than -0.02eV/Å and the change in energy smaller than 10^-5^ eV was attained. Grimme’s DFT-D3 methodology was used to describe the dispersion interactions among all the atoms in adsorption models. The Gibbs free energy (ΔG) of reaction intermediates was calculated by the following:

ΔG = ΔE + ΔE_ZPE_−TΔS

where ΔE is the adsorption energy. ΔE_ZPE_ and ΔS are the difference for the zero-point energy and entropy, respectively. The zero-point energy and entropy were calculated at the standard conditions corresponding to the pressure of 101325 Pa (~1 bar) of H_2_ at the temperature of 298.15 K. The climbing image nudged elastic band (CINEB) method was used to search the reaction path and transition state, and the vibration frequency calculation was used to confirm it further.

3. Figures and Tables

**
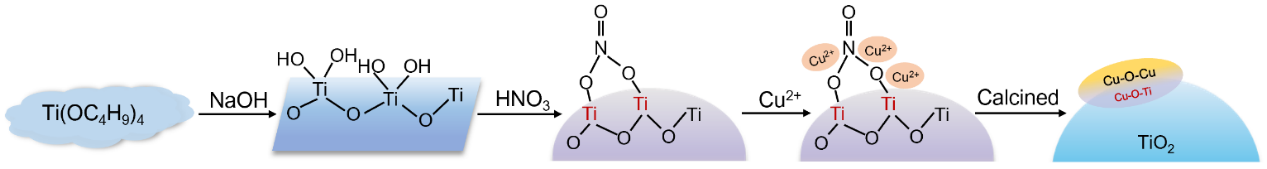
**

Figure S1. Schematic illustration of the preparation method.

**
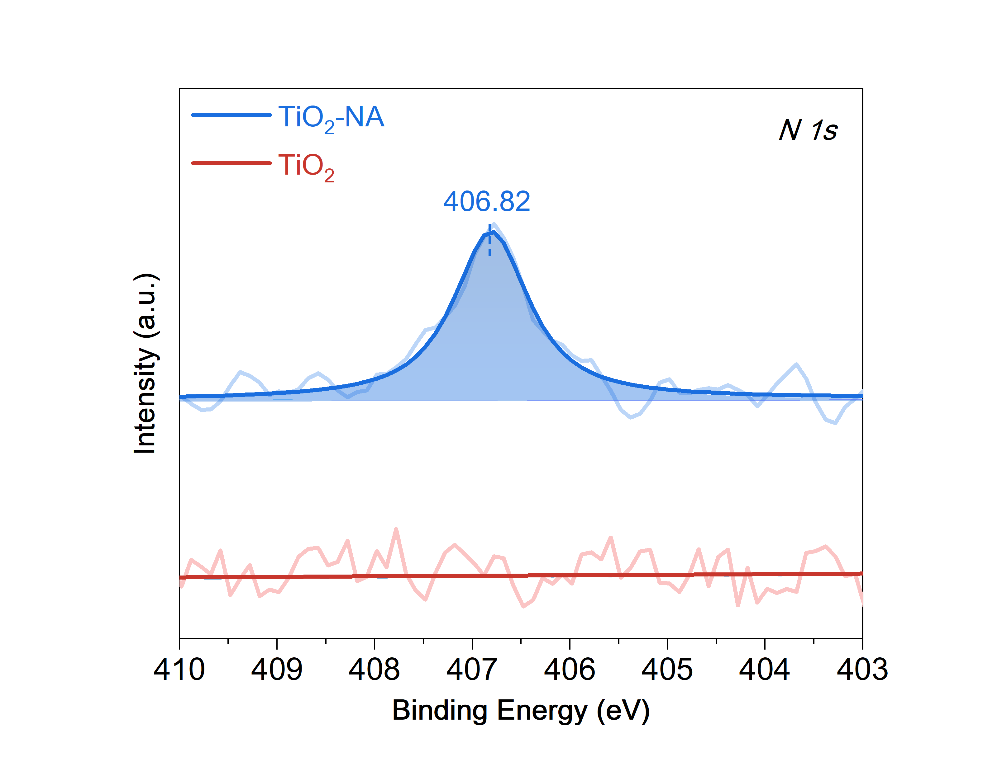
**

Figure S2. N 1s XPS spectrum of TiO_2_-NA and TiO_2_.

The N 1s XPS spectrum of TiO_2_-NA exhibits a noticeable peak at 406.82 eV related to NO_3_^-^, which is absent in TiO_2_.


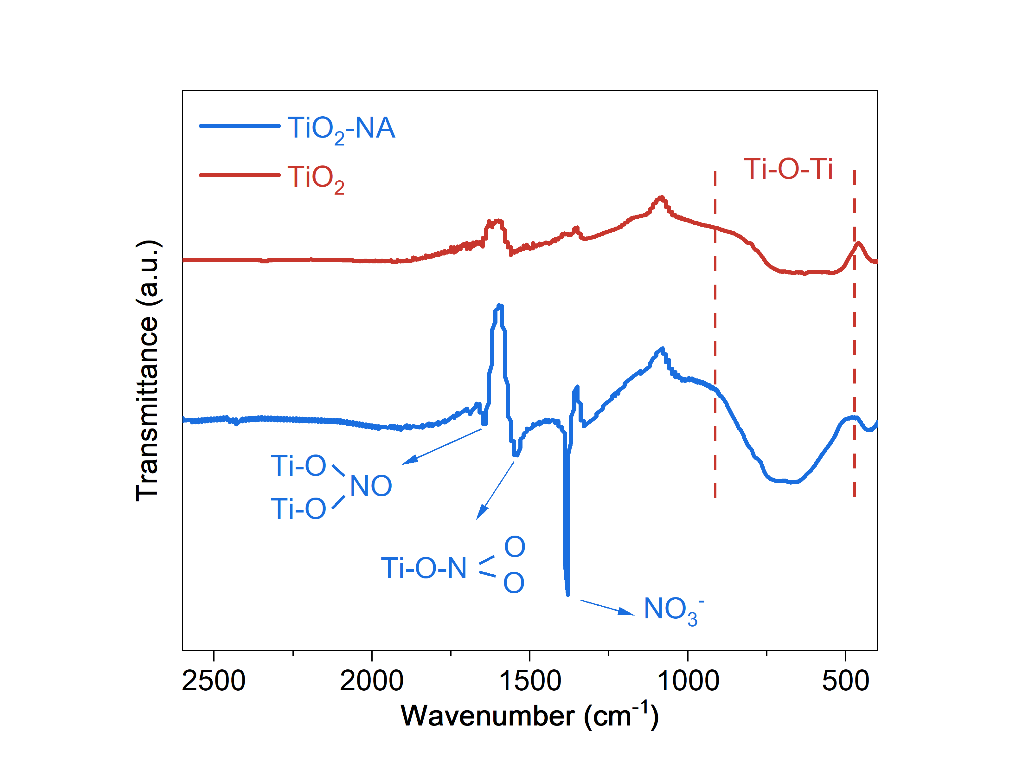


Figure S3. FT-IR spectra of TiO_2_-NA and TiO_2_.

Fourier transform infrared spectroscopy (FT-IR) of TiO_2_-NA and TiO_2_ further confirms the nitrate groups grafted successfully by the presence of nitrate and corresponding ester bonds.


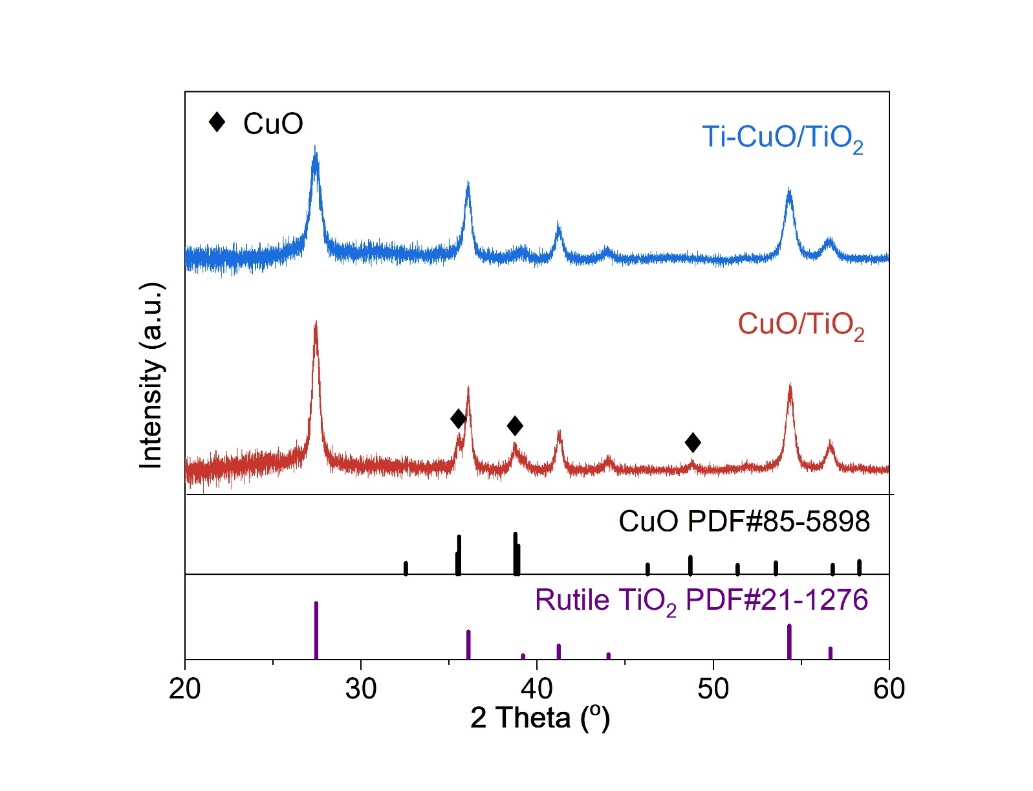


Figure S4. XRD patterns of Ti-CuO/TiO_2_ and CuO/TiO_2_.


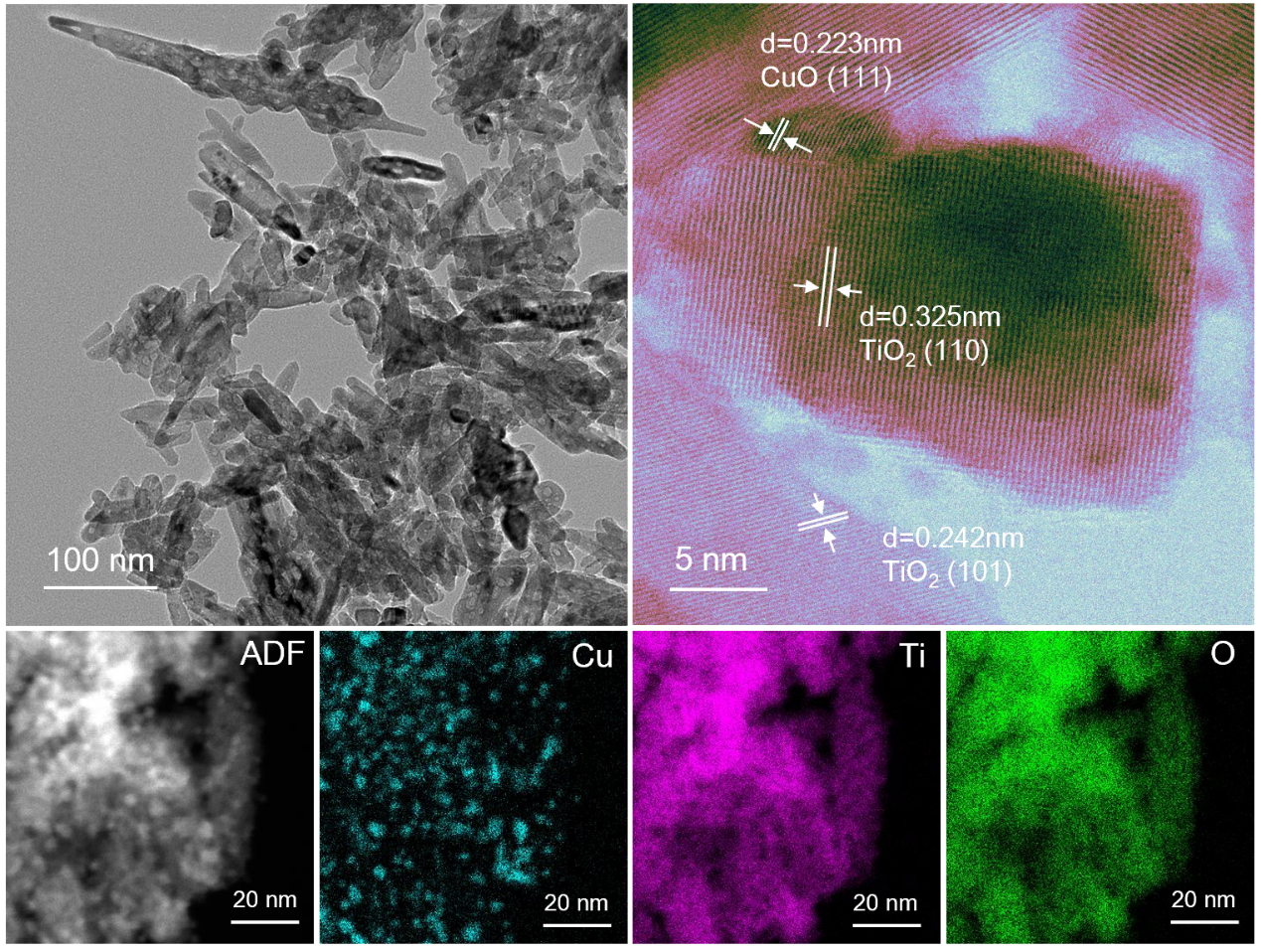


Figure S5. HAADF-STEM images with the corresponding EDS mappings for Ti-CuO/TiO_2_.

**
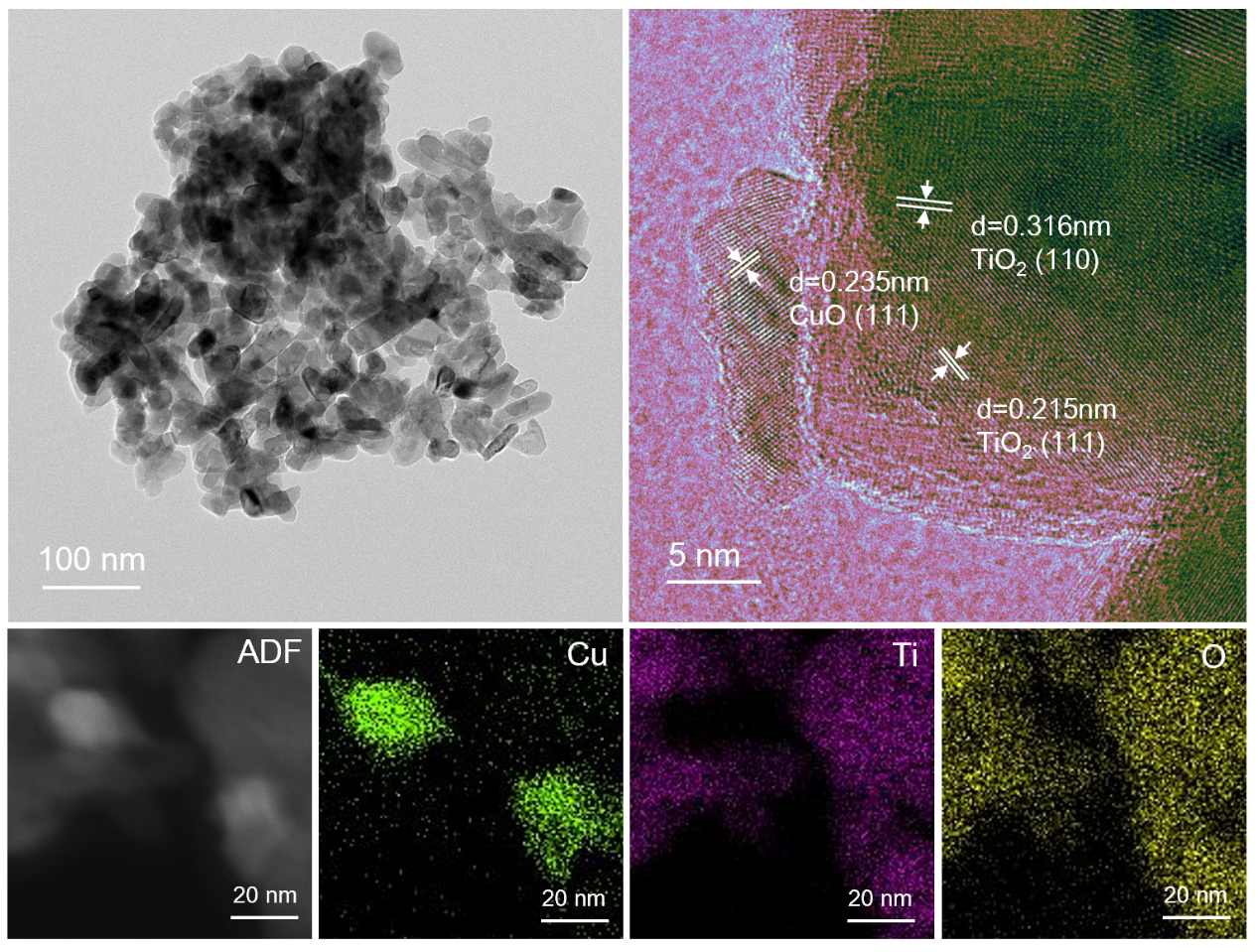
**

Figure S6. HAADF-STEM images with the corresponding EDS mappings for CuO/TiO_2_.

**
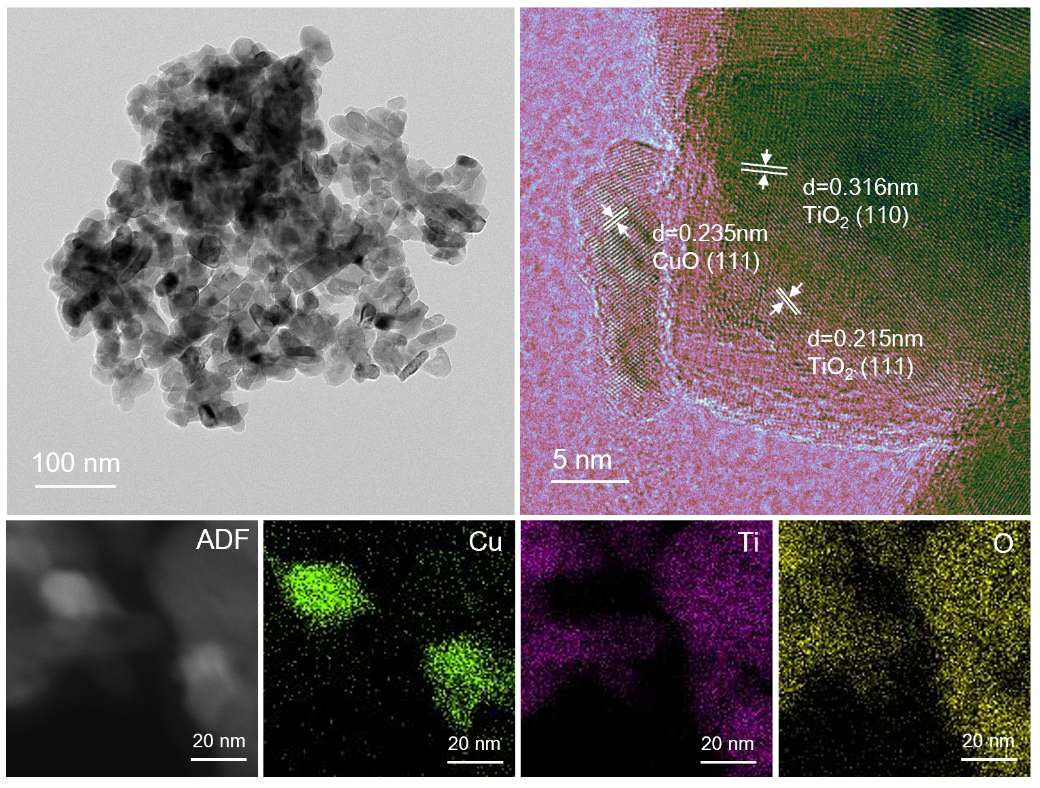
**

Figure S7. HRTEM images for CuO/TiO_2_.


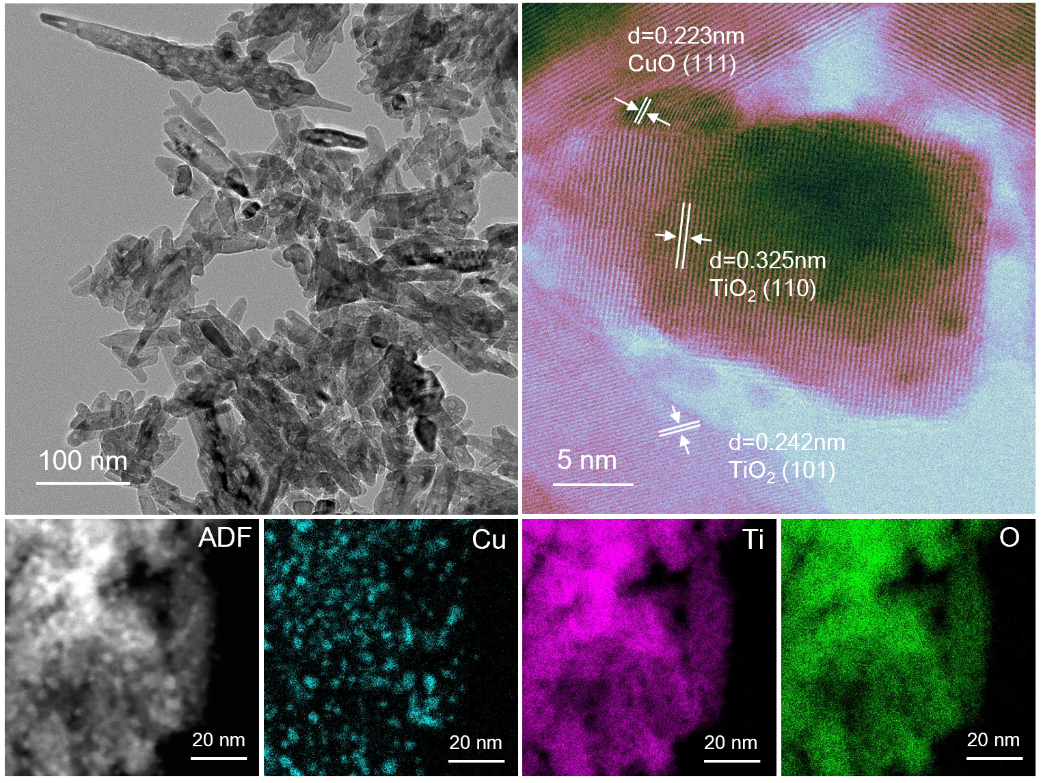


Figure S8. HRTEM images for Ti-CuO/TiO_2_.


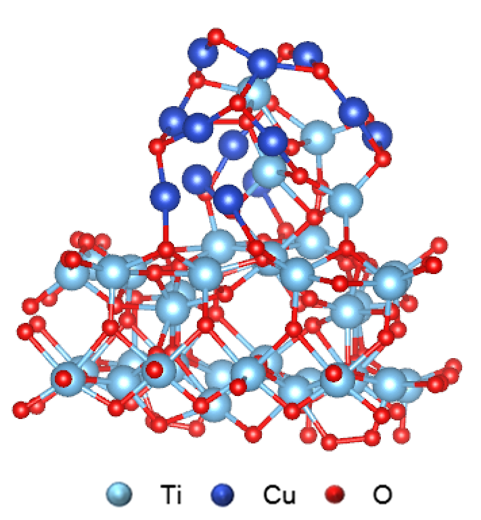


Figure S9. Optimized model for Ti-CuO/TiO_2_ catalyst.

Based on the HRTEM images, Ti-CuO(111)/TiO_2_(110) structure is optimized as the computational models for Ti-CuO/TiO_2_. From the oxygen vacancy formation energy of Cu-O-Cu and Ti-O-Cu results, the oxygen vacancy is also constructed on Cu-O-Ti of Ti-CuO(111)/TiO_2_(110).


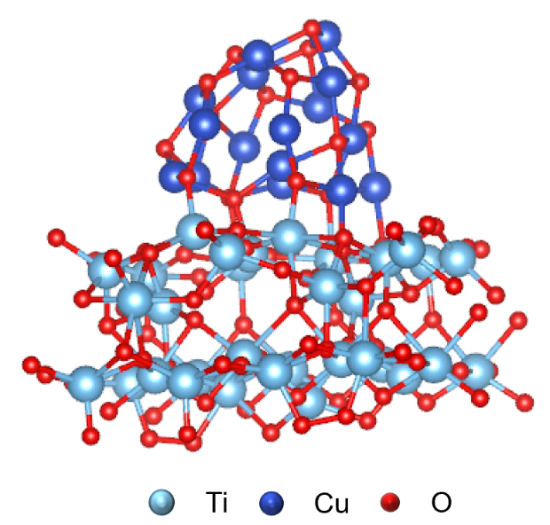


Figure S10. Optimized model for CuO/TiO_2_ catalyst.

Based on the HRTEM images, the CuO(111)/TiO_2_(110) structure is optimized as the computational models for CuO/TiO_2_.


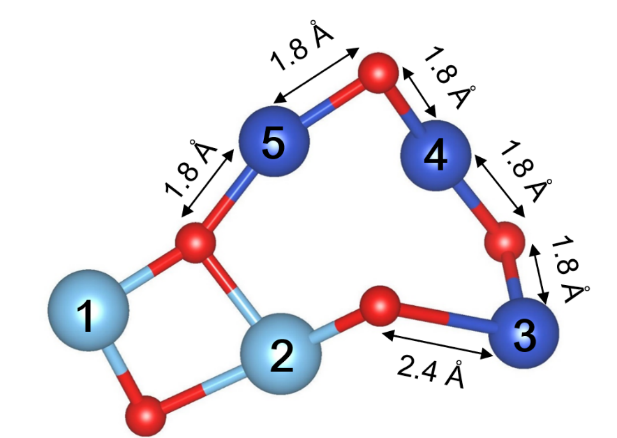


Figure S11. Bond length of Cu-O in Ti-O-Cu-O-Cu-O-Cu structure.


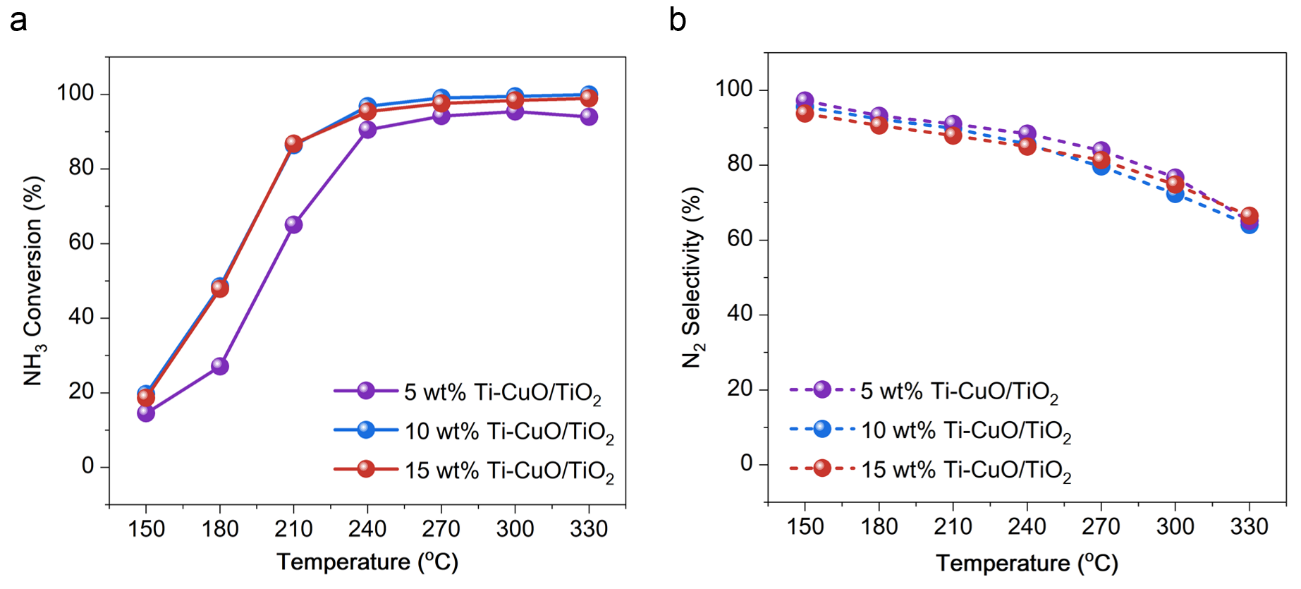


Figure S12. NH_3_ conversion (a) and N_2_ selectivity (b) as a function of temperature of Ti-CuO/TiO_2_ catalysts with the CuO loading of 5 wt%, 10 wt%, and 15 wt%. Conditions: [NH_3_] = 500 ppm, [O_2_] = 5 vol%, N_2_ as balance and GHSV = 50,000 h^-1^.

**
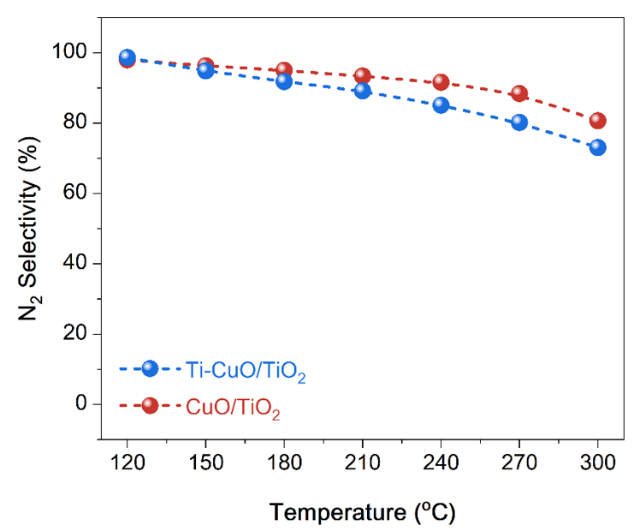
**

Figure S13. N_2_ selectivity as a function of temperature of Ti-CuO/TiO_2_ and CuO/TiO_2_. Conditions: [NH_3_] = 500 ppm, [O_2_] = 5 vol%, N_2_ as balance and GHSV = 50,000 h^-1^.


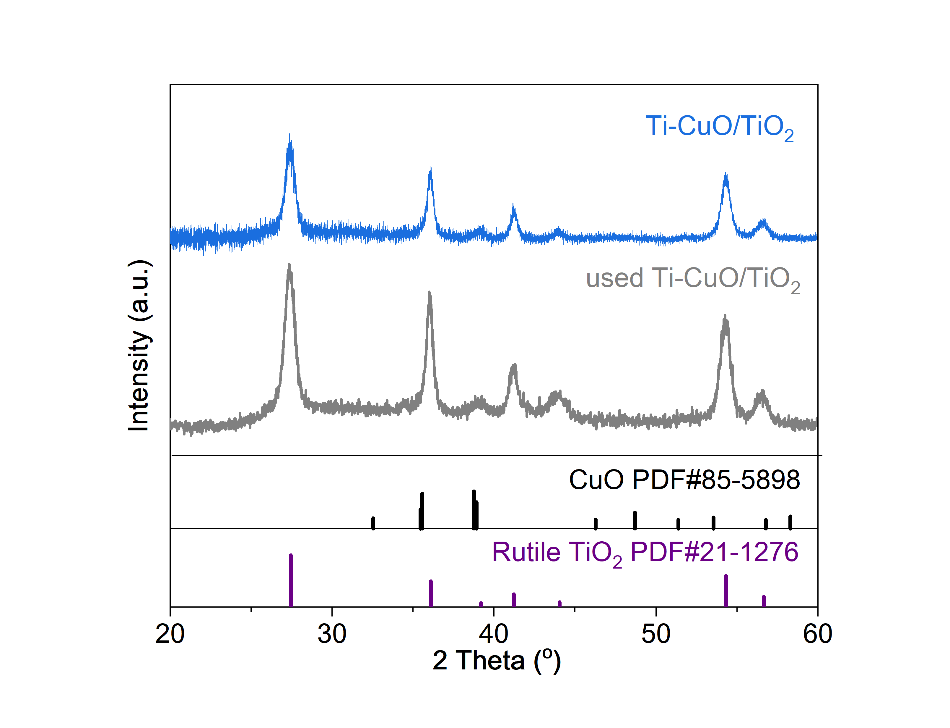


Figure S14. XRD patterns of Ti-CuO/TiO_2_ and Ti-CuO/TiO_2_ after the durability test.

The XRD pattern of used Ti-CuO/TiO_2_ shows only the characteristic peaks of the rutile TiO_2_ support (JCPDS No. 21-1276), with no detectable diffraction peaks corresponding to crystalline CuO.


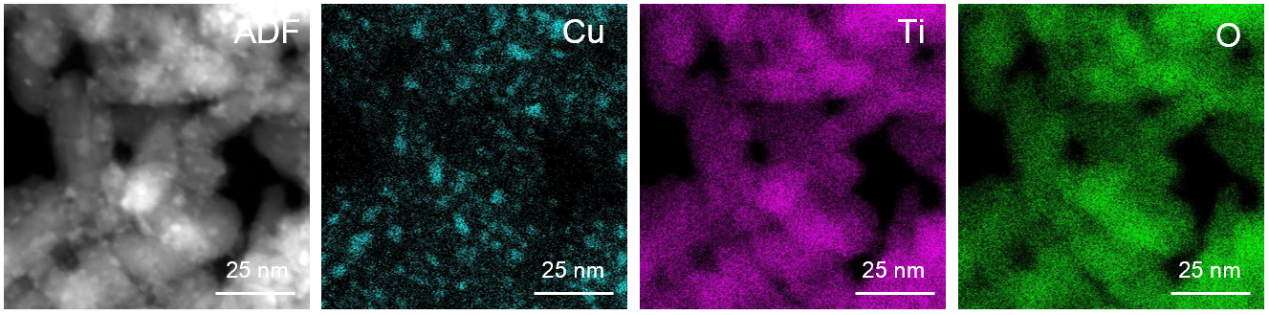


Figure S15. HAADF-STEM images with the corresponding EDS mappings for Ti-CuO/TiO_2_ after the durability test.

The CuO nanoparticles remain uniformly distributed on Ti-CuO/TiO_2_ after the durability test, with no visible agglomeration compared to the fresh catalyst (Figure S5)


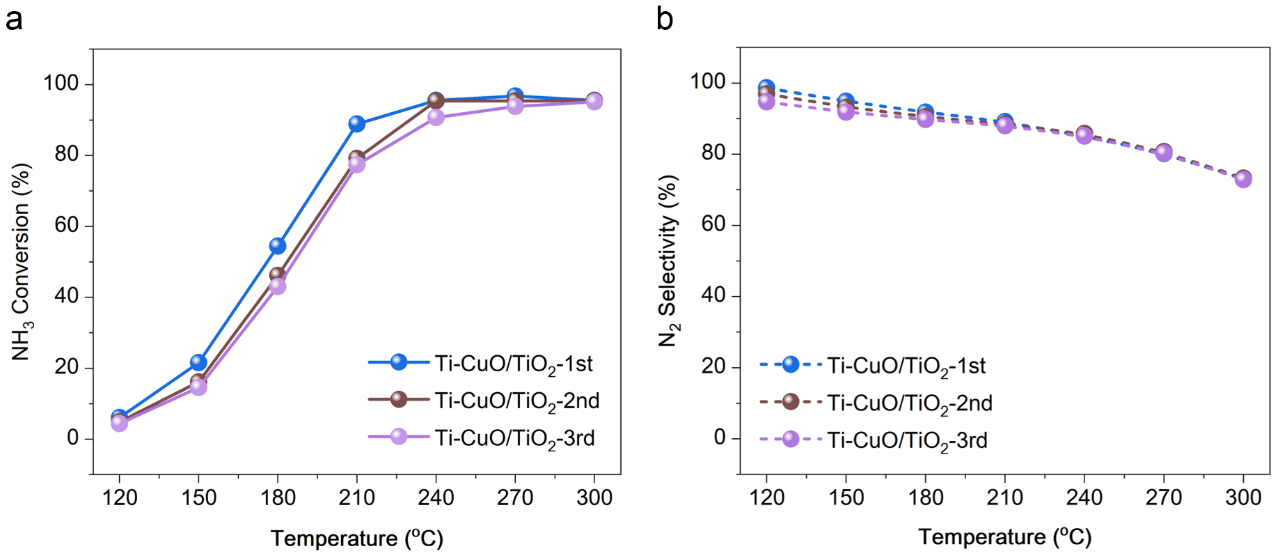


Figure S16. The cycling NH_3_-SCO performance. NH_3_ conversion (a) and N_2_ selectivity (b) of Ti-CuO/TiO_2_ catalyst. Conditions: [NH_3_] = 500 ppm, [O_2_] = 5 vol%, N_2_ as balance and GHSV= 50,000 h^-1^.


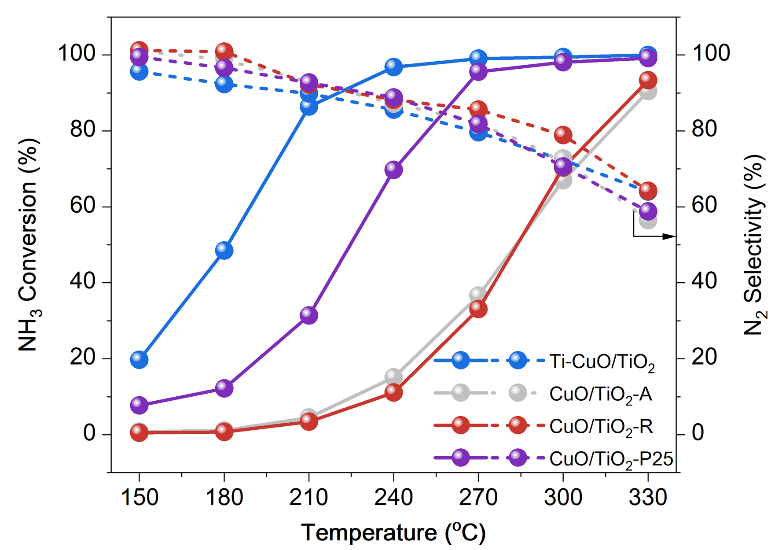


Figure S17. NH_3_ conversion and N_2_ selectivity as a function of temperature of Ti-CuO/TiO_2_, CuO/TiO_2_-A, CuO/TiO_2_-R and CuO/TiO_2_-P25. Conditions: [NH_3_] = 500 ppm, [O_2_] = 5 vol%, N_2_ as balance and GHSV = 50,000 h^-1^.


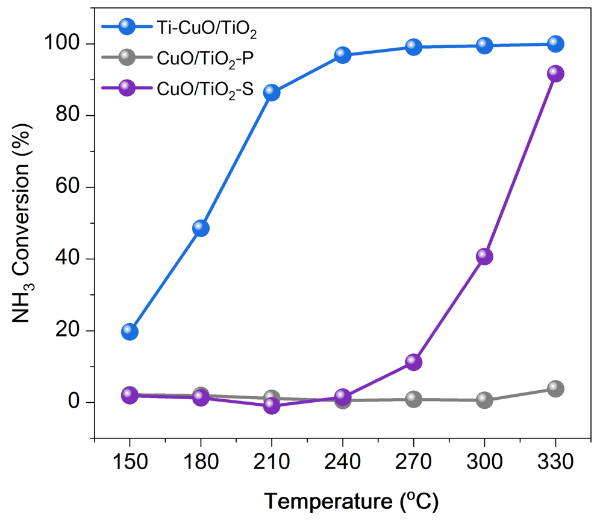


Figure S18. NH_3_ conversion as a function of temperature of Ti-CuO/TiO_2_, CuO/TiO_2_-P and CuO/TiO_2_-S. Conditions: [NH_3_] = 500 ppm, [O_2_] = 5 vol%, N_2_ as balance and GHSV = 50,000 h^-1^.


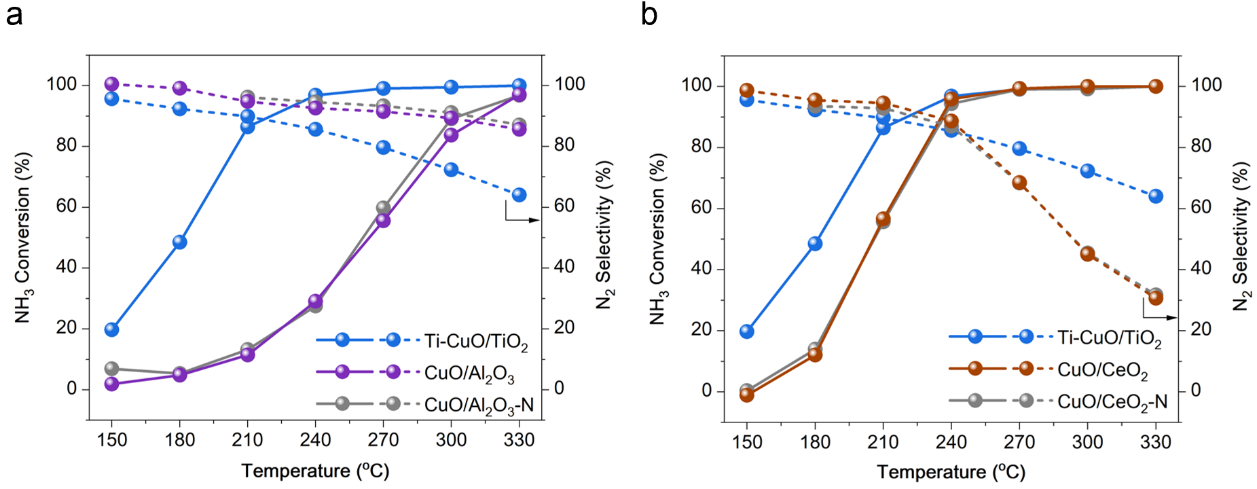


Figure S19. NH_3_ conversion and N_2_ selectivity as a function of temperature of Ti-CuO/TiO_2_, CuO/Al_2_O_3_ and CuO/ Al_2_O_3_-N (a) and Ti-CuO/TiO_2_, CuO/CeO_2_ and CuO/ CeO_2_-N (b). Conditions: [NH_3_] = 500 ppm, [O_2_] = 5 vol%, N_2_ as balance and GHSV = 50,000 h^-1^.


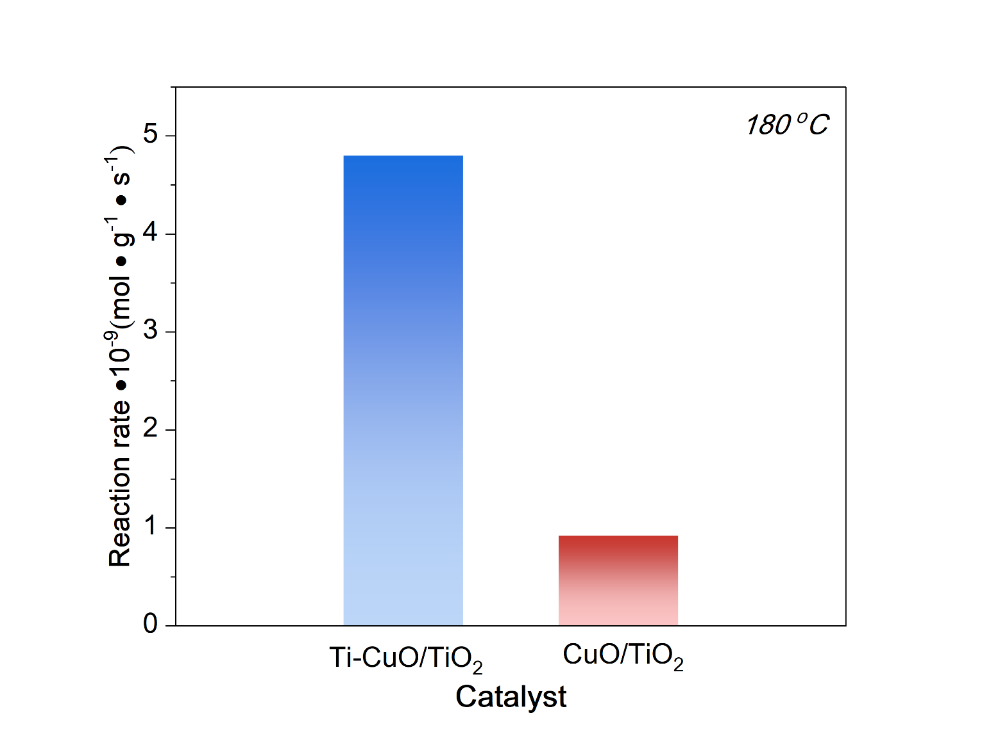


Figure S20. Reaction rates at 180 °C of Ti-CuO/TiO_2_ and CuO/TiO_2_.


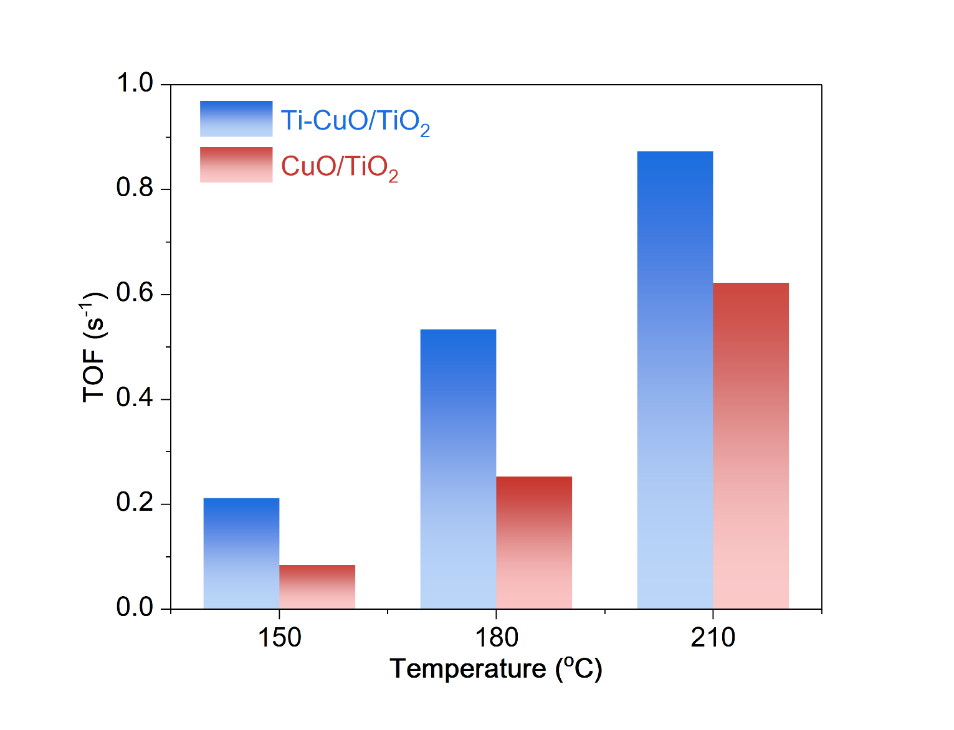


Figure S21. TOF of Ti-CuO/TiO_2_ and CuO/TiO_2_ at temperatures of 150°C, 180°C, and 210°C.


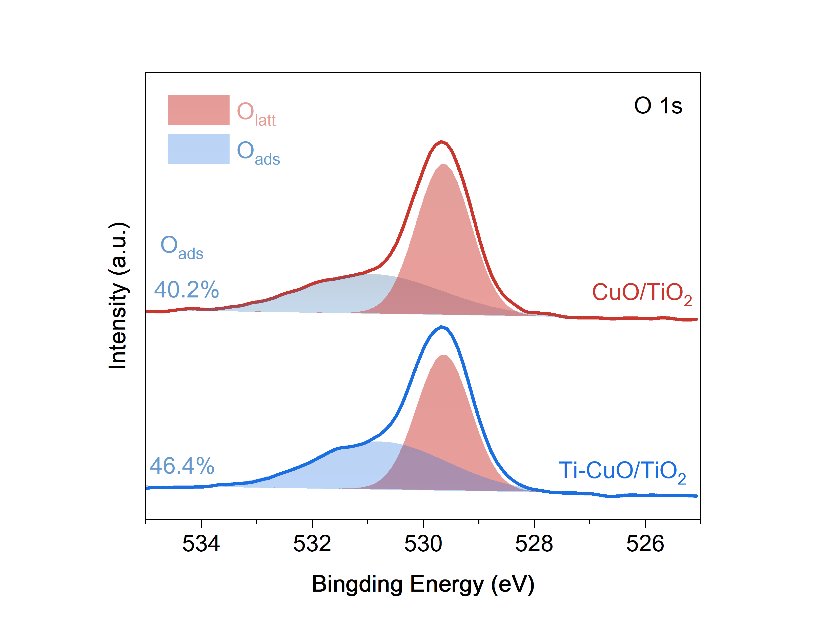


Figure S22. O 1s XPS spectra of Ti-CuO/TiO_2_ and CuO/TiO_2_.

The O 1s XPS spectra of catalysts show the surface adsorbed oxygen (denoted as O_ads_) and lattice oxygen (denoted as O_latt_) species at the binding energies of 530.9 and 529.6 eV, respectively.^[1]^ Ti-CuO/TiO_2_ shows more surface adsorption oxygen species that are considered as the reactive oxygen species for oxidation reaction.


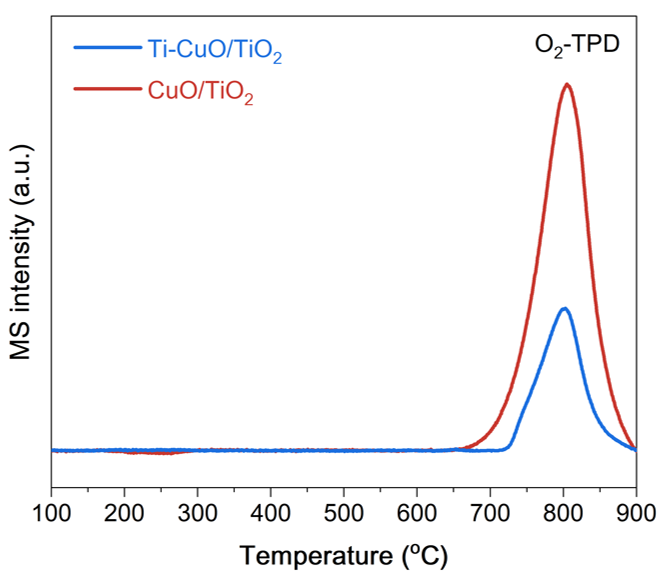


Figure S23. MS signals in O_2_-TPD of Ti-CuO/TiO_2_ and CuO/TiO_2_.

Both catalysts show a broad desorption peak in the 700-900 °C range in O_2_-TPD, which is attributed to the thermal decomposition of crystalline CuO into Cu_2_O and gaseous O_2_.^[2]^ The lower peak intensity of Ti-CuO/TiO_2_ indicates the reduced crystallinity of CuO and higher oxygen mobility.


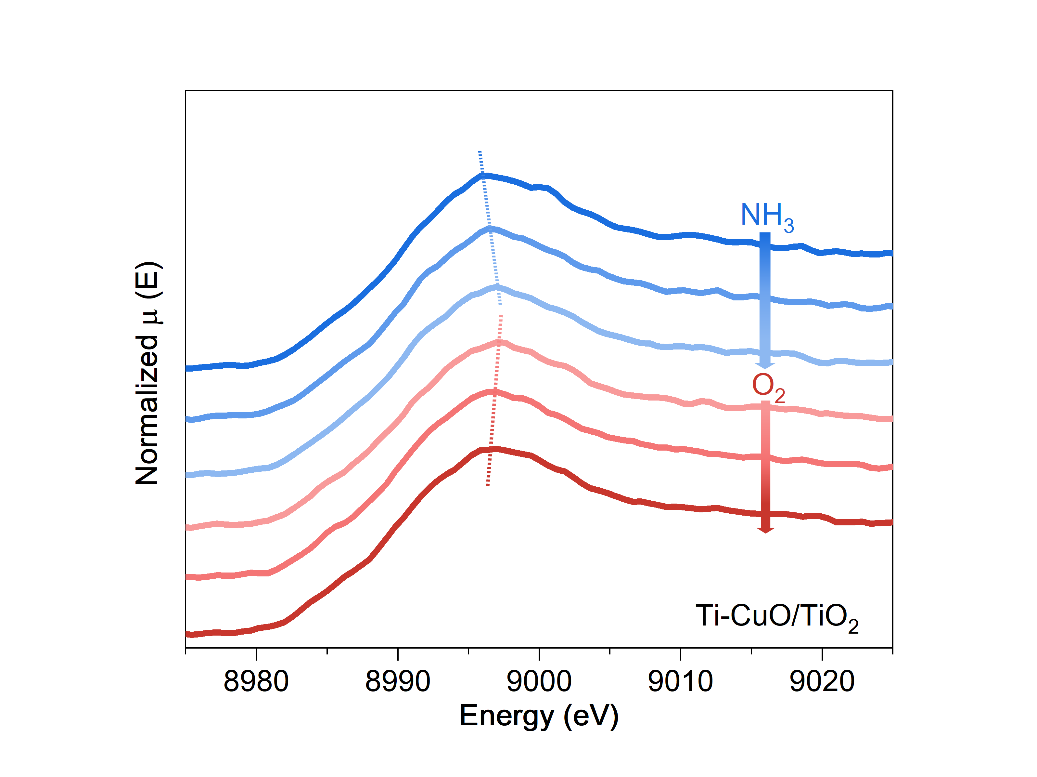


Figure S24. *In situ* Cu K-edge XANES spectra of the transient reaction between O_2_ and pre-adsorbed NH_3_ at 200 ^o^C of Ti-CuO/TiO_2_.


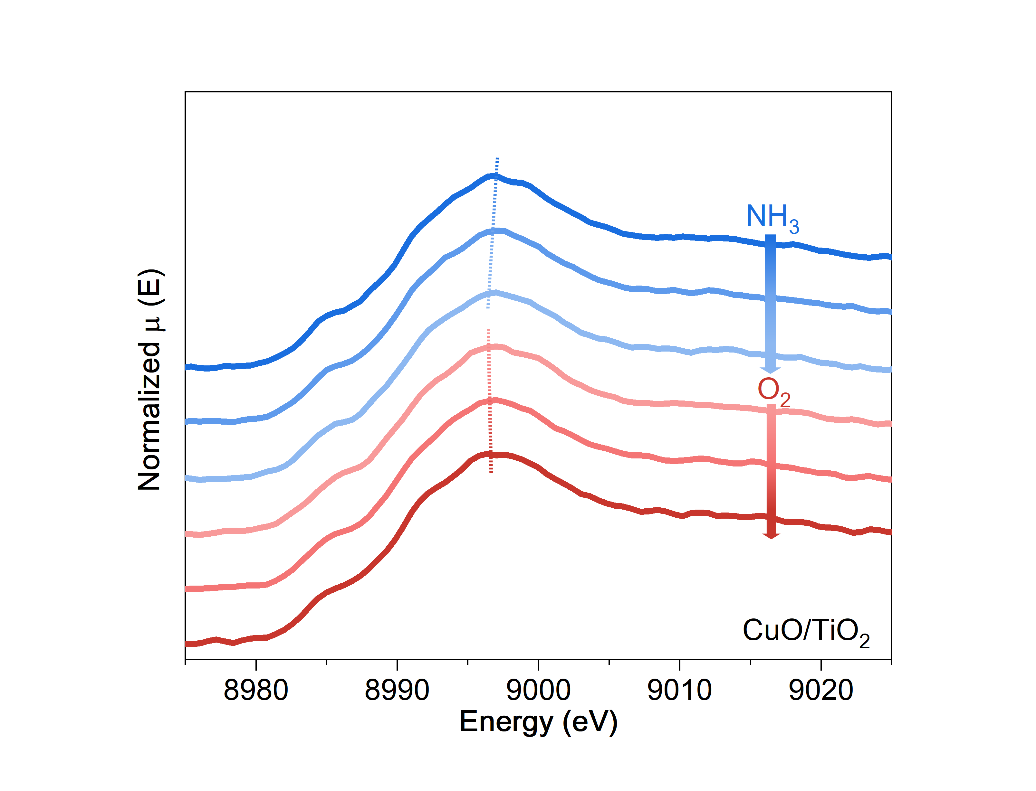


Figure S25. *In situ* Cu K-edge XANES spectra of the transient reaction between O_2_ and pre-adsorbed NH_3_ at 200 ^o^C of CuO/TiO_2_.


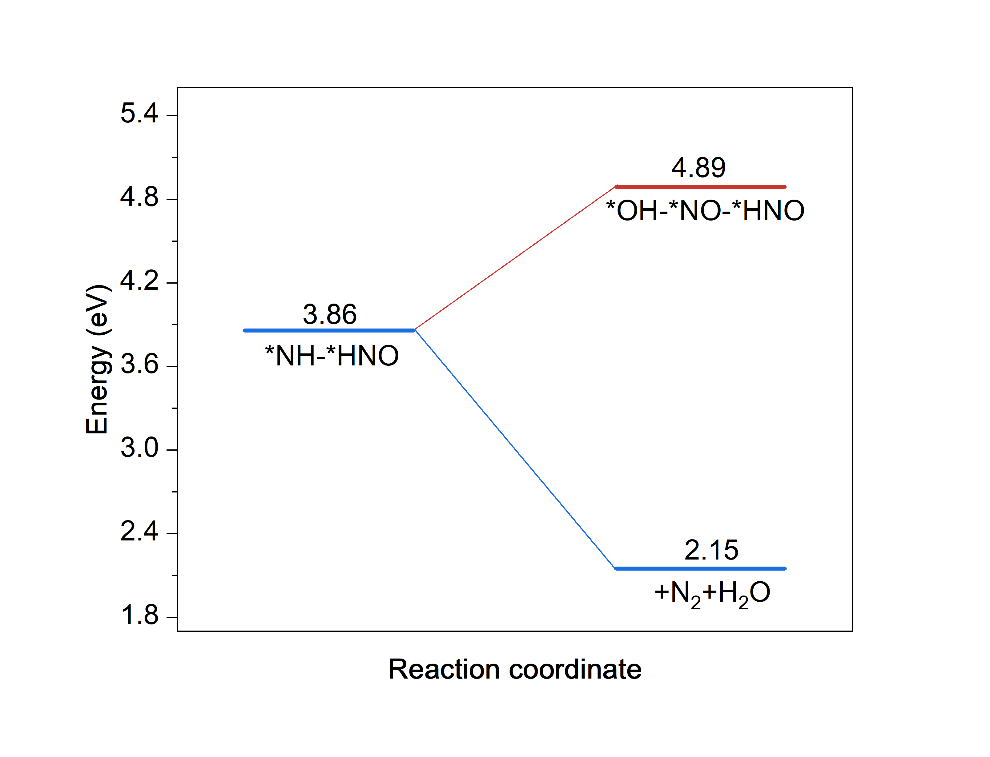


Figure S26. Calculated energy of *NH before and after reacting with *O (pink) and *HNO (blue) on Ti-CuO/TiO_2_.

Table S1. The result of dissociative N_2_O oxidation and H_2_ titration for Ti-CuO/TiO_2_ and CuO/TiO_2_.

| **Catalyst** | **Hydrogen Consumption (mmol/g)** | | **D_Cu_** |
| --- | --- | --- | --- |
|  | **A_1_** | **A_2_** | **2A_2_ / A_1_** |
| Ti-CuO/TiO_2_ | 1.68 | 0.37 | 0.44 |
| CuO/TiO_2_ | 1.44 | 0.15 | 0.21 |

Table S2. Relative area proportion of Cu^2+^ and Cu^+^ in XPS for Ti-CuO/TiO_2_ and CuO/TiO_2_.

| **Catalyst** | **Relative area proportion** | |
| --- | --- | --- |
|  | **Cu^2+^/ΣCu (%)** | **Cu^+^/ΣCu (%)** |
| Ti-CuO/TiO_2_ | 61.2 | 38.8 |
| CuO/TiO_2_ | 69.0 | 31.0 |

Table S3. Fit parameters of EXAFS data of Ti-CuO/TiO_2_ catalyst.

| **Catalyst** | **Shell** | **CN** | **R(Å)** | **σ^2^ (Å^2^)** | **ΔE_0_(eV)** |
| --- | --- | --- | --- | --- | --- |
| Ti-CuO/TiO_2_ | Cu-O | 4.1±0.2 | 1.97±0.01 | 0.003 | -0.04±0.8 |
|  | Cu-O-Ti | 1.0±0.2 | 2.41±0.02 | 0.010 | -0.04±0.8 |
|  | Cu-O-Cu | 1.7±0.4 | 2.96±0.02 | 0.010 | -0.04±0.8 |

CN is the coordination number; R is the distance between absorber and backscatter atoms; σ^2^ is the Debye-Waller factor to account for both thermal and structural disorders; ΔE_0_ is the inner potential correction (edge-energy shift).

Table S4. Fit parameters of EXAFS data of CuO/TiO_2_ catalyst.

| **Catalyst** | **Shell** | **CN** | **R(Å)** | **σ^2^ (Å^2^)** | **ΔE_0_(eV)** |
| --- | --- | --- | --- | --- | --- |
| CuO/TiO_2_ | Cu-O | 4.0±0.7 | 1.95±0.02 | 0.004 | 0.5±1.9 |
|  | Cu-O-Cu | 3.0±0.8 | 2.95±0.02 | 0.004 | 0.5±1.9 |

Table S5. Copper and CuO contents in Ti-CuO/TiO_2_ from ICP-OES.

| **Catalyst** | **Cu (%)** | **CuO (%)** | **Average (%)** |
| --- | --- | --- | --- |
| Ti-CuO/TiO_2_ | 7.88 | 9.86 | 9.84 |
|  | 7.78 | 9.74 |  |
|  | 7.92 | 9.91 |  |

Table S6. Comparison upon NH_3_-SCO performance of the catalyst in this work with the reported catalysts.

| **Catalyst** | **T_50_, T_90_ (℃)** | **N_2_ Sel. at T_90_（%）** | **Reaction conditions** | **Ref** |
| --- | --- | --- | --- | --- |
| **Ti-CuO/TiO_2_** | **180, 210** | **90** | **500 ppm NH_3_, 5% O_2_, 60000 h^-1^** | **This work** |
| CuO_x_/La_2_Ce_2_O_7_ | 197, 243 | 85 | 500 ppm NH_3_, 5% O_2_, 20000 h^-1^ | [3] |
| CuO_x_/Al_2_O_3_-H | 215, 240 | 85 | 500 ppm NH_3_, 5% O_2_, 60000 h^-1^ | [4] |
| CuO(2%)/TiO_2_ | 240, 275 | 90 | 500 ppm NH_3_, 5% O_2_, 60000 h^-1^ | [5] |
| Cu_S_(4%)/ZSM-5 | 305, 345 | 90 | 5000 ppm NH_3_, 5% O_2_, 120000 mL∙g^-1^∙ h^-1^ | [6] |
| Cu(2%)/NCNT | 186, 212 | 85 | 500 ppm NH_3_, 1% O_2_, 120000 mL∙g^-1^∙ h^-1^ | [7] |
| CuO(20%)/CeO_2_ | 192, 221 | 96 | 1000 ppm NH_3_, 10% O_2_, 40000 h^-1^ | [8] |
| Fe_2_O_3_-SiO_2_ | 350, 450 | 65 | 1000 ppm NH_3_, 2% O_2_, 200000 h^-1^ | [9] |
| MnO_x_-TiO_2_ | 160, 210 | 80 | 500 ppm NH_3_, 5% O_2_, 240000 mL∙g^-1^∙ h^-1^ | [10] |
| Pt/Al_2_O_3_ | 210, 230 | 78 | 1000 ppm NH_3_, 10 % O_2_, 1000 mL/min | [11] |
| 1%Pt/C9Z1 | 210, 250 | 53 | 500 ppm NH_3_, 1 % O_2_, 200000 mL∙g^-1^∙ h^-1^ | [12] |
| 10%Ag/Al_2_O_3_ | 208, 220 | 80 | 500 ppm NH_3_, 5 % O_2_, 75000 h^-1^ | [13] |
| Pt/Al_2_O_3_@Cu/ZSM-5 | 250, 284 | 92 | 500 ppm NH_3_, 1% O_2_, 120000 mL∙g^-1^∙ h^-1^ | [14] |

Table S7. Specific surface area (S_BET_) and pore properties of Ti-CuO/TiO_2_ and CuO/TiO_2_.

| **Catalyst** | **S_BET_**  **m^2^∙g^-1^** | **Pore diameter**  **nm** | **Pore volume**  **cm^3^∙g^-1^** |
| --- | --- | --- | --- |
| Ti-CuO/TiO_2_ | 46.44 | 28.06 | 0.355 |
| CuO/TiO_2_ | 31.10 | 23.17 | 0.191 |

Table S8. Fit parameters of *in-situ* EXAFS data of the transient reaction between O_2_ and pre-adsorbed NH_3_ at 200 ^o^C of Ti-CuO/TiO_2_.

| **Measurement** | **Shell** | **CN** | **R(Å)** | **σ^2^ (Å^2^)** | **ΔE_0_(eV)** |
| --- | --- | --- | --- | --- | --- |
| N_2_ pretreatment | Cu-N/O | 4.2±0.7 | 1.95±0.02 | 0.009 | -0.2±1.9 |
|  | Cu-O-Ti | 0.8±0.5 | 2.37±0.04 | 0.012 | -0.2±1.9 |
|  | Cu-O-Cu | 1.9±0.3 | 2.93±0.03 | 0.012 | -0.2±1.9 |
| NH_3_ introduced | Cu-N/O | 5.1±1.5 | 1.92±0.06 | 0.013 | -4.2±4.6 |
|  | Cu-O-Cu | 1.5±0.5 | 2.86±0.03 | 0.006 | -4.2±4.6 |
| O_2_ introduced | Cu-N/O | 4.5±0.8 | 1.96±0.01 | 0.009 | -0.5±1.6 |
|  | Cu-O-Ti | 0.8±0.3 | 2.36±0.03 | 0.010 | -0.5±1.6 |
|  | Cu-O-Cu | 2.1±0.6 | 2.94±0.02 | 0.010 | -0.5±1.6 |

Table S9. Fit parameters of *in-situ* EXAFS data of the transient reaction between O_2_ and pre-adsorbed NH_3_ at 200 ^o^C of CuO/TiO_2_.

| **Measurement** | **Shell** | **CN** | **R(Å)** | **σ^2^ (Å^2^)** | **ΔE_0_(eV)** |
| --- | --- | --- | --- | --- | --- |
| N_2_ pretreatment | Cu-N/O | 4.9±0.9 | 1.93±0.02 | 0.009 | -2.9±2.1 |
|  | Cu-Cu1 | 1.9±1.0 | 2.80±0.05 | 0.007 | -2.9±2.1 |
|  | Cu-Cu2 | 2.4±1.1 | 2.98±0.05 | 0.007 | -2.9±2.1 |
| NH_3_ introduced | Cu-N/O | 4.2±0.4 | 1.94±0.02 | 0.006 | -2.7±2.2 |
|  | Cu-Cu1 | 2.1±1.1 | 2.81±0.05 | 0.006 | -2.7±2.2 |
|  | Cu-Cu2 | 2.6±1.2 | 2.99±0.05 | 0.006 | -2.7±2.2 |
| O_2_ introduced | Cu-N/O | 4.4±1.1 | 1.94±0.02 | 0.007 | -2.7±2.6 |
|  | Cu-Cu1 | 2.2±1.3 | 2.82±0.04 | 0.007 | -2.7±2.6 |
|  | Cu-Cu2 | 2.8±1.4 | 2.99±0.06 | 0.007 | -2.7±2.6 |

**References**

[1] L. Han, M. Gao, C. Feng, L. Shi, D. Zhang, *Environ. Sci. Technol.* **2019**, *53*, 5946-5956.

[2] J. Shen, Z. Qiao, J. Wang, G. Yang, J. Chen, Z. Li, X. Liao, H. Wang, M. R. Zachariah, *Thermochim. Acta.* **2018**, *666*, 60-65.

[3] X. Kong, Z. Li, Y. Shao, X. Ren, K. Li, H. Wu, C. Lv, C. Lv, S. Zhu, *J. Mater. Sci. Technol.* **2021**, *111*, 1-8.

[4] T. Lan, J. Deng, X. Zhang, F. Wang, X. Liu, D. Cheng, D. Zhang, *ACS Catal.* **2022**, *12*, 3955-3964.

[5] M. Ran, Y. Dong, X. Zhang, W. Li, Z. Wang, S. Lin, Y. Yang, H. Song, W. Wu, S. Liu, Y. Zhu, C. Zheng, X. Gao, *Environ. Sci. Technol.* **2024**, *58*, 12249-12259.

[6] L. Chen, X. Guan, X. Wu, H. Asakura, D. G. Hopkinson, C. Allen, J. Callison, P. J. Dyson, F. R. Wang, *Proc. Natl. Acad. Sci.* **2024**, *121*, e2404830121.

[7] L. Peng, A. Guo, D. Chen, P. Liu, B. Peng, M. Fu, D. Ye, P. Chen, *Environ. Sci. Technol.* **2022**, *56*, 14008-14018.

[8] H. Sun, H. Wang, Z. Qu, *ACS Catal.* **2023**, *13*, 1077-1088.

[9] R. Long, R. Yang, *J. Catal.* **2002**, *207*, 158-165.

[10] D. Song, X. Shao, M. Yuan, L. Wang, W. Zhan, Y. Guo, Y. Guo, G. Lu, *RSC Adv.* **2016**, *6*, 88117-88125.

[11] R. S. Ghosh, P. S. Dhillon, M. P. Harold, D. Wang, *Chem. Eng. J.* **2020**, *417*, 128273.

[12] W. Tan, Y. Cai, H. Yu, S. Xie, M. Wang, K. Ye, L. Ma, S. N. Ehrlich, F. Gao, L. Dong, F. Liu, *Environ. Sci. Technol.* **2023**, *57*, 15747-15758.

[13] G. Xu, Y. Zhang, J. Lin, Y. Wang, X. Shi, Y. Yu, H. He, *ACS Catal.* **2021**, *11*, 5506-5516.

[14] R. S. Ghosh, T. T. Le, T. Terlier, J. D. Rimer, M. P. Harold, D. Wang, *ACS Catal.* **2020**, *10*, 3604-3617.
